# Integrative Roles of Phytohormones on Cell Proliferation, Elongation and Differentiation in the *Arabidopsis thaliana* Primary Root

**DOI:** 10.3389/fpls.2021.659155

**Published:** 2021-04-26

**Authors:** Estephania Zluhan-Martínez, Brenda Anabel López-Ruíz, Mónica L. García-Gómez, Berenice García-Ponce, María de la Paz Sánchez, Elena R. Álvarez-Buylla, Adriana Garay-Arroyo

**Affiliations:** ^1^Laboratorio de Genética Molecular, Desarrollo y Evolución de Plantas, Departamento de Ecología Funcional, Instituto de Ecología, Universidad Nacional Autónoma de México, Ciudad de México, Mexico; ^2^Centro de Ciencias de la Complejidad, Universidad Nacional Autónoma de México, Ciudad de México, Mexico

**Keywords:** hormones, Arabidopsis, root apical meristem, primary root, cell proliferation, cell elongation, cell differentiation

## Abstract

The growth of multicellular organisms relies on cell proliferation, elongation and differentiation that are tightly regulated throughout development by internal and external stimuli. The plasticity of a growth response largely depends on the capacity of the organism to adjust the ratio between cell proliferation and cell differentiation. The primary root of *Arabidopsis thaliana* offers many advantages toward understanding growth homeostasis as root cells are continuously produced and move from cell proliferation to elongation and differentiation that are processes spatially separated and could be studied along the longitudinal axis. Hormones fine tune plant growth responses and a huge amount of information has been recently generated on the role of these compounds in Arabidopsis primary root development. In this review, we summarized the participation of nine hormones in the regulation of the different zones and domains of the Arabidopsis primary root. In some cases, we found synergism between hormones that function either positively or negatively in proliferation, elongation or differentiation. Intriguingly, there are other cases where the interaction between hormones exhibits unexpected results. Future analysis on the molecular mechanisms underlying crosstalk hormone action in specific zones and domains will unravel their coordination over PR development.

## Introduction

**FIGURE 1:**
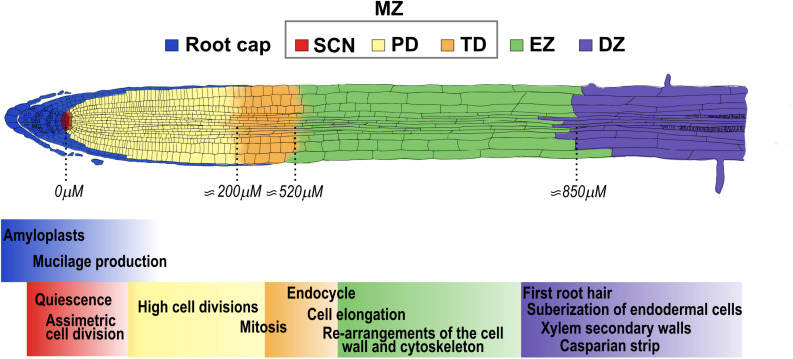
Arabidopsis primary root developmental zones and domains. The root is composed from its tip along the longitudinal axis by the stem cell niche (SCN-red) that consists of stem cells that surround the Quiescent Center. The primary root is divided in three developmental zones: the meristematic zone (MZ), that includes the SCN, the proliferation domain (PD-yellow) and the transition domain (TD-orange); the elongation zone (EZ-green) and the differentiation zone (DZ-purple). The root cap (blue) surrounds the tip of the root until the end of the MZ. The approximate distance from the QC to the end of the PD, TD and EZ at 5 days after germination (5dag) WT seedlings is indicated below (Verbelen et al., 2006). The cellular functions that occur in each of the developmental zones and domains of the root are highlighted with the color that corresponds to each developmental stage.

Plant development depends on three interlinked processes: cell proliferation, elongation and differentiation, that can be studied *in vivo* in *Arabidopsis thaliana* (hereafter Arabidopsis) roots. Roots are an excellent model to study the balance between cell division, elongation and differentiation as these processes are spatially separated along the main axis (Scheres and Wolkenfelt, 1998). The organization from the tip of the root to the base of the stem consists of different tissues and zones, starting with the columella, which confers soil abrasion resistance, followed by the MZ, the Elongation Zone (EZ) and the Differentiation Zone (DZ) (Figure 1; Baluška et al., 1996; Verbelen et al., 2006; Ivanov and Dubrovsky, 2013; Salvi et al., 2020). The MZ is the region within the root where cells are produced, and it consists of the Stem Cell Niche (SCN), the Proliferation Domain (PD) and the Transition Domain (TD) (Baluška et al., 1994, 2010; Verbelen et al., 2006; Ivanov and Dubrovsky, 2013; Salvi et al., 2020; Figure 1). The SCN has a central organizer known as the Quiescent Center (QC) surrounded by stem cells that divide to self-renew and to provide cells that will populate either the PD or the columella, in the case of the distal stem cells (Dolan et al., 1993; Van den Berg et al., 1995; Van Den Berg et al., 1997; Benfey and Scheres, 2000; Figure 1). In the MZ, the cells divide 4 to 6 times and then they transit to the EZ where there is a rapid longitudinal expansion, until eventually the cells reach the DZ where they acquire their final characteristics (Dolan et al., 1993; Barrada et al., 2015; Figure 1). In addition, the primary root (PR) of Arabidopsis has a very simple radial organization with cell types arranged around the innermost vascular tissues; the epidermis is the most external layer, followed by the cortex, the 40 endodermis, the pericycle and the vascular tissues in the center. The lateral root cap protects the 41 epidermis at the very root tip, and it is only present in the Meristematic Zone (MZ) (Dolan et al., 1993).

Roots are essential for anchorage, water and nutrients uptake, for the establishment of symbiotic associations with different organisms and for environmental sensing (Hodge, 2009). Therefore, root development is highly plastic in response to an ever-changing environment where the hormones participate in all plant developmental processes and exhibit cell types, organs and tissues-specific responses (Gray, 2004; Hodge, 2009; Sánchez-Calderón et al., 2013). Information regarding the biosynthesis, conjugation, transport, catabolism, perception and signal transduction pathways of auxin, abscisic acid (ABA), brassinosteroid (BR), strigolactone (SL), gibberellic acid (GA), cytokinin (CK), jasmonic acid (JA), salicylic acid (SA) and ethylene have been widely studied, and details were summarized in Supplementary Figure 1 and Supplementary Tables 1, 2. Moreover, the participation of them in Arabidopsis PR development has been widely studied either in mutants of loss or gain-of-function (LoF or GoF; Supplementary Table 3 to see the root phenotype); by treatments with chemicals that change their concentration or distribution as well as by using hormonal-response gene constructs (Tanimoto, 2005; Jung and McCouch, 2013; Qin et al., 2019; Waidmann et al., 2020). Not surprisingly, the function of each hormone is spatially regulated and depends specifically on the organ and the plant developmental stage (Wang and Irving, 2011). Despite the huge amount of information generated in the last two decades on the effect of hormones in PR growth, their participation in all domains and zones have still not been fully integrated (Takatsuka and Umeda, 2014). Hormones regulate cell division and cell elongation in Arabidopsis roots and the length of either the MZ and the fully elongated cells, are two sensitive and quantitative parameters to evaluate their participation in PR growth (Rahman et al., 2000; Okamoto et al., 2008; Tapia-López et al., 2008; Garay-Arroyo et al., 2013; Chaiwanon et al., 2016; Moubayidin et al., 2016; Cajero Sánchez et al., 2018). To shed light on this matter, in this review we summarize and discuss the information regarding the effect of diverse hormones in each developmental zone that comprise the PR.

### Hormone Function in the Meristematic Zone

As mentioned above, the MZ consists of two different domains: the proliferation domain (PD) and the transition domain (TD) (Verbelen et al., 2006; Ivanov and Dubrovsky, 2013; Salvi et al., 2020; Figure 1).

#### Hormone Control of Cell Division in the Proliferation Domain

**FIGURE 2:**
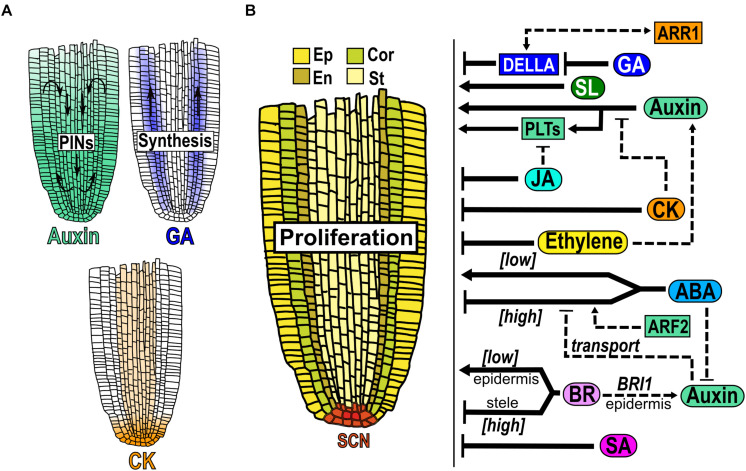
Hormone function in the proliferation domain. **(A)** Hormones accumulation in the MZ. The auxin gradient (green) is established by the transport network formed by PIN efflux proteins and GA (blue) produced in the PD moves upwards this zone. GA is synthesized mainly in cortex and endodermis cells and CK (light orange) in vascular tissue. **(B)** Structure of the PD: the stem cell niche (SCN red) at the base produces cells that are incorporated to the PD where they proliferate at a high rate in either the epidermis (Ep), cortex (Cor), endodermis (En) and stele (St). At the right of the Figure is depicted the hormonal regulation (black arrows) and crosstalk (dotted arrows) over the MZ growth.

As the proliferation domain (PD) is where most cells are produced (Ivanov and Dubrovsky, 2013; García-Gómez et al., 2017; Figure 2B) and most studies do not differentiate between the PD and the TD and treat them as the MZ, we will interpret the reported meristematic data as PD unless otherwise is indicated.

In the root auxin, GA and SL are hormones that promote cell division in the MZ. Regarding auxin, it has a maximum concentration in the QC cells where it is synthesized, high in the PD and relatively low in the EZ and the DZ (Blilou et al., 2005; Vieten et al., 2005; Petersson et al., 2009; Brunoud et al., 2012; Band et al., 2014). Application of low auxin levels (200 nM IAA) stimulate mitotic activity resulting in a larger MZ (Růzička et al., 2009; Figure 2B). In addition, the double LoF mutant in the auxin biosynthetic genes *WEI8*/*TAA1* and *TAR2* (*wei8 tar2*) does not have an identifiable MZ and the treatment with IAA, partially re-establish the meristem (Brumos et al., 2018). Auxin distribution in the MZ depends mainly on the action of the PIN-FORMED (PIN) proteins that are auxin efflux carriers differentially distributed in root tissues and this, in turn, regulates MZ size (Blilou et al., 2005; Vieten et al., 2005; Figure 2A). PIN proteins have phosphorylation-dependent polar localization in the plasma membrane and the overexpression of the protein kinase *PINOID* (*PID*) induces a basal-to-apical shift in PIN1, PIN2 and PIN4 localization in the MZ cells, altering the gradient of auxin and triggering a collapse of the MZ (Friml et al., 2004). A similar phenotype has been seen in the LoF of the protein phosphatase 2A (*PP2A*) that regulates apical-to-basal PIN cell localization (Michniewicz et al., 2007). The redundant function of the PIN proteins makes their analyses difficult and the single LoF mutants of *PIN* genes (*PIN1*, *2*, *3*, *4* and *7*) hardly affect MZ size while the double and triple mutants diminish it (Blilou et al., 2005). Contrary to the plasma membrane PIN proteins, the LoF mutants of *PIN5* and *PIN-LIKES 6* (*PILS6*), that are auxin carriers localized in the endoplasmic reticulum, have enlarged meristems; accordingly, the OE of anyone of both genes, have shorter meristems compared to WT (Mravec et al., 2009; Di Mambro et al., 2019; Feraru et al., 2019).

*PLETHORA (PLT)* genes are transcription factors (TF) induced and regulated downstream of the auxin signaling pathway and function in a dose-dependent manner to maintain cell proliferation in the PD (Galinha et al., 2007; Mähönen et al., 2014; Santuari et al., 2016; Scheres and Krizek, 2018; Figure 2B). *PLT1*, *PLT2*, *PLT3* and *PLT4* (*BABY BOOM*, *BBM*) are expressed in the COL, SCN and PD, their proteins form gradients all over the PR and function redundantly to control meristem size (Galinha et al., 2007; Mähönen et al., 2014). Also, high levels of *PLT2* are related with slow division rates in the SCN, intermediate levels are required to maintain high cell division in the MZ, and low levels promote cell expansion and differentiation; in addition, the OE of *PLT2* has a larger MZ (Galinha et al., 2007; Mähönen et al., 2014). The double mutant of *PLT1* and *PLT2* has a short MZ and the quadruple mutant (*PLT1*, *PLT2*, *PLT3* and *BBM*) is rootless (Aida et al., 2004; Galinha et al., 2007).

Besides, *MONOPTEROS* (*MP)/ARF5*, an auxin response factor, positively regulates the expression of miR390, which mediates the auxin responses in the MZ (Dastidar et al., 2019); its LoF mutant is rootless while the weak *mp/arf5* allele (*mpS319)* has short MZ in postembryonic development (Berleth and Jurgens, 1993; Konishi et al., 2015). Furthermore, the auxin signaling repressor *IAA3/SHORT HYPOCOTYL 2 (SHY2)* regulates the size of the MZ, since the GoF mutant of this gene (*shy2-2)* has a reduced MZ with less cells, while the LoF mutant (*shy2-31*) has a larger MZ than WT (Dello Ioio et al., 2008b; Moubayidin et al., 2010).

GA is produced in the meristem where the cortex and the endodermis are important sites of its biosynthesis (Figure 2A). This hormone is required for cell division and the EZ requires GA production or action from the MZ for cell elongation (Barker et al., 2020; Figure 2B). Besides, GA is a promoter of cell proliferation and addition of the biological active GA, increases the size of the MZ whereas the treatment with paclobutrazol (PAC), an inhibitor of GA synthesis, results in a reduced MZ (Ubeda-Tomás et al., 2009; Moubayidin et al., 2010, 2016; Figure 2B). GA modulates the MZ size via a DELLA dependent mechanism as the GA-deficient mutant *ga1-3* displays a reduction in MZ size which can be reverted when crossed with the quadruple LoF mutant of the negative regulators of GA signal transduction, the DELLAs *GIBBERELLIC ACID INSENSITIVE* (*GAI*), *REPRESSOR OF GA1-3* (*RGA*), *RGA-LIKE1* (*RGL1*), and *RGL2* (*gai-t6 rga-t2 rgl1-1 rgl2-1*) (Figure 2B). Indeed, *GAI* transcript accumulation in the endodermis of the MZ is sufficient to reduce the MZ size partially by enhancing the levels of two cell cycle inhibitors: *KIP-RELATING PROTEIN 2* (*KRP2*) and *SIAMESE* (*SIM*) (Ubeda-Tomás et al., 2008, 2009; Achard et al., 2009). The expression of the TF *SCARECROW* (*SCR*) in the endodermis of the LoF *scr-1* mutant, partially reestablishes the MZ size of the mutant (Sabatini, 2003). Interestingly, GA application increases the MZ size in WT plants but not in the *scr* mutant, showing that SCR is required to mediate this GA effect (Moubayidin et al., 2016). Additionally, epistatic genetic analysis showed that *RGA* and *SCR* participate in different signaling pathways controlling MZ size despite the fact that SCR controls the stability of this DELLA protein (Moubayidin et al., 2016).

In addition, treatment with the synthetic SL analog GR24 (2.5μM) increases PD size, compared to untreated plants (Ruyter-Spira et al., 2011; Figure 2B). Besides, the SL signaling mutant of *MORE AXILLARY GROWTH 2* (*max2*), the SL biosynthesis mutants *max1* and *CAROTENOID CLEAVAGE DIOXYGENASE 8* (*max4*), have shorter meristems than WT plants (Ruyter-Spira et al., 2011).

Conversely, CK, ethylene, JA and SA are hormones that inhibit cell division in the PD/MZ. CK concentration is lower in the MZ compared to the SCN (Antoniadi et al., 2015; Figure 2A). This hormone negatively controls the MZ size and positively the meristematic cell differentiation in a dose-dependent manner (Dello Ioio et al., 2007; Ishida et al., 2010; Takahashi et al., 2013; Street et al., 2015; Figure 2B). Diminishing CK levels and/or signaling results in a larger MZ and a delay in the onset of endoreplication as exemplified by the triple LoF mutant of isopentenyl transferase enzymes (IPTs) (*ipt3 ipt5 ipt7*) and in mutants affecting either the CK signal transduction pathway (*ARABIDOPSIS HISTIDINE KINASE 3/4* (*AHK3/4*) or type B *ARR1/12*) or by causing an increase in CK catabolism by the OE of *CYTOKININ OXIDASE/DEHYDROGENASE* (*CKX*) (Werner et al., 2003; Dello Ioio et al., 2007; Ishida et al., 2010; Takahashi et al., 2013; Street et al., 2016). Moreover, the double mutant (*phb phv*) of the LoF of *PHABULOSA* (*PHB*) and *PHAVOLUTA* (*PHV*), that are members of the TFs HD-ZIPII family, has a longer meristem than WT plants in control conditions and the CK treatment restores the root phenotype to WT (Dello Ioio et al., 2012).

Ethylene, as CK, negatively regulates the MZ size in a dose-dependent manner (Street et al., 2015; Zdarska et al., 2019; Figure 2B). Furthermore, ethylene reduces the activity of a cell cycle reporter (*CYCB1;1-GUS*) in the PD, which is reverted by the co-treatment with an ethylene inhibitor (1-methylcyclopropene, 1-MCP). Interestingly, *CYCB1;1* expression is not affected in response to ethylene, suggesting post-transcriptional regulation (Street et al., 2015). In addition, the LoF of the *CONSTITUTIVE TRIPLE RESPONSE 1* (*CTR1-2*), a negative regulator of ethylene pathway, has a shorter MZ. Consistently, the LoF mutant *etr1-1*, an ethylene insensitive mutant, has longer MZ than WT plants (Street et al., 2015; Méndez-Bravo et al., 2019; Zdarska et al., 2019).

JA treatment reduces the MZ size in a *COI1*/*MYC2* dependent manner (Figure 2B) by repressing the expression of cell cycle genes like *CYCB1;1*, *CYCLIN DEPENDENT KINASE A;1* (*CDKA;1)*, *KRP1* and *PROLIFERATING CELL NUCLEAR ANTIGEN* (*PCNA1)* (Chen et al., 2011). Similarly, SA treatment reduces the MZ size (Figure 2B) and the expression of *CYCB1;1* (Pasternak et al., 2019).

Additionally, ABA and BR can either promote or inhibit PD/MZ activity in a dose-dependent fashion. Low exogenous ABA concentrations (0.5 μM) increases the MZ size (Zhang et al., 2010), whereas high concentrations (30 μM) inhibit it compared to control conditions (Yang et al., 2014; Figure 2B). Interestingly, the MZ size inhibition by ABA can be partially recovered by the co-treatment with the reducing agent glutathione (GSH), suggesting that ABA controls the MZ size through reactive oxygen species (ROS) regulation (Yang et al., 2014). Curiously, it has been reported that local application of ABA to the shoot, in a concentration that when applied to the roots inhibits MZ size (2 μM), positively regulates the MZ size compared to control conditions (Xie et al., 2020). This ABA effect is mediated by the long-range transport of auxin from the shoot to the root, that promotes cell division in the MZ (Xie et al., 2020).

Plants treated with high concentrations of 0.4–4 nM of BR, have a shorter MZ than untreated plants, whilst 0.04nM of BR increase the MZ size (González-García et al., 2011; Chaiwanon and Wang, 2015; Li et al., 2020; Figure 2B). Accordingly, low BR concentrations (50 pM) could rescue MZ length whereas higher concentrations (1 nM and 100 nM) reduce the MZ size in the BR-deficient mutant *dwf4* (González-García et al., 2011; Chaiwanon and Wang, 2015). Also, low endogenous BR levels or high BR response that can be obtained either with mutations in BR biosynthesis and signaling genes (*de*-*etiolated 2* (*det2-1*), *bri1-5, BES1-RNAi* or the GoF mutant *bin2-*1) or with the GoF of *BRI1-EMS-SUPPRESSOR 1* (*BES1; bes1-D*), with enhanced BR signaling, lead to plants with shorter MZ than WT (González-García et al., 2011; Hacham et al., 2011). Concordantly, the triple LoF mutant of the negative regulator *BRASSINOSTEROID INSENSITIVE 2* (*BIN2)* and its two homologs *BIN2-LIKE1* (*BIL1)* and *BIL2* (*bin2-3 bil1 bil2*) show a larger MZ (González-García et al., 2011; Li et al., 2020). In addition, the BR-insensitive mutant *bri1-116*, has a shorter MZ than WT, due to a decrease in the cell cycle progression that can be reverted to WT plants in the double mutant with the OE of *CYCD3;1* (*bri1-116 CYCD3;1*OE) (González-García et al., 2011; Hacham et al., 2011). Interestingly, the BR effect over the meristem cell proliferation or cell differentiation depends on the cell type where it is active: in the epidermis, BR signaling is necessary to induce the number of proliferating cells whilst in the stele it promotes cell differentiation (Hacham et al., 2011; Vragović et al., 2015). Furthermore, the MZ length of the single mutant of the receptor *BRASSINOSTEROID INSENSITIVE 1* (*BRI1; bri1*) and the triple mutant of *BRI1* with *BRI1 LIKE1* (*BRL1*) and *BRL3* (*bri1 brl1 brl3*) are bigger than WT if *BRI1* is expressed in the epidermal non-hair-cells (Hacham et al., 2011; Vragović et al., 2015; Figure 2B). Also, the expression of the GoF of *BRASSINAZOLE RESISTANT 1* (*BZR1; bzr1-1D*) or *BRI1* in the epidermis in the *bri1* LoF mutant background can rescue the MZ size (Chaiwanon and Wang, 2015). Contrarily, the expression in the stele of *BRI1* counteracts the proliferation induced by epidermal *BRI1* expression (Vragović et al., 2015; Figure 2B). All these data suggest the importance not only of BR dosage but also the cell type where it is active to control the MZ size.

The CK and auxin crosstalk is well described, and it is known to regulate the MZ size by controlling the transition from proliferation to elongation (Salvi et al., 2020). CK interferes with auxin responses and transport in the MZ as follow: *SHY2/IAA3* is positively regulated by ARR1 and is sufficient to mediate ARR1 function in the MZ size, because the OE of *ARR1* in the LoF mutant *shy2-31*, does not show a MZ reduction. In addition, the double mutants, *arr1-3 shy2-2* and *arr1-3 shy2-31* have the same MZ size than the single *arr1-3* mutant (Dello Ioio et al., 2008b). Moreover, SHY2 in turn, negatively regulates the expression of the efflux auxin transporters (PINs) (Dello Ioio et al., 2008b). Besides, the MZ size of the double and triple auxin influx transport with CK signaling mutants, *aux1 arr12* and *aux1 arr1 arr12*, is higher than *aux1* single mutant and WT plants but identical to the single *arr12* or the double *arr1 arr12* CK signaling mutants, suggesting that CK is epistatic to auxin in this phenotype (Street et al., 2016; Figure 2B) (more details on CK-auxin crosstalk are also described in the TD section). Additionally, the tryptophan (Trp) synthesis gene *ANTHRANILATE SYNTHASE B1 (ASB1)*, which is expressed in the MZ, is also regulated by CK via ARR1 (Moubayidin et al., 2013).

JA negatively regulates the expression of *PLT1* and *PLT2* genes through the direct repression by the TF *MYC2* (Figure 2B). Interestingly, the double mutant *plt1-4 plt2-2* is less inhibited by JA application than WT, but the hormone still reduces the MZ size of this mutant (Chen et al., 2011) suggesting the existence of also a PLT-dependent mechanism by which JA regulates the meristem.

CK participates in the ethylene inhibition of the MZ size as the LoF mutants of the CK TFs, *ARR1* and *ARR3*, are less responsive to ethylene inhibition of MZ size than WT plants and it has been described that CK induces ethylene biosynthesis (Chae et al., 2003; Street et al., 2015; Zdarska et al., 2019). Interestingly, the ethylene-insensitive mutants (*etr1-1* and *ein2-1*) reduce the MZ responsiveness only at low CK concentrations (0.1 and 1 μm) but not at high CK levels (10μm) (Street et al., 2015). Moreover, the co-treatment with an ethylene inhibitor (1-MCP) partially alleviates the CK inhibition of the MZ size compared to plants only treated with CK, confirming a role for ethylene in cytokinin response in the MZ size (Street et al., 2015; Figure 2B).

Compared to CK and JA, that negatively impact on auxin function in the MZ, ethylene enhances local IAA production (Figure 2B) by promoting the transcript accumulation of two genes that participate in auxin biosynthesis, *WEI8/TAA1* and *TAR2* (Stepanova et al., 2008). Moreover, ethylene application or the LoF of *CTR1*, stimulates *AUX1* and *PIN2* expression and promotes auxin movement throughout the PD, especially from the root tip to the TD and EZ. This auxin re-distribution is not observed in the auxin transport (*aux1)* and synthesis *(wei2* and *wei7)* mutants, where the co-treatment with ethylene promotes auxin accumulation strictly limited to cells of the COL and part of the PD (Stepanova et al., 2005, 2007; Rùžièka et al., 2007; Swarup et al., 2007; Méndez-Bravo et al., 2019; Figure 2B). In addition, the *pin2* LoF mutant has a longer MZ, but the double mutant *ctr1-1eir1-1*(*pin2*) has a total absence of the MZ (Méndez-Bravo et al., 2019).

Regarding auxin and its crosstalk with the biphasic hormones that participate in the MZ, it has been shown that the induction of cell proliferation in the MZ caused by *BRI1* expression in epidermis is achieved by increasing the auxin concentration in this zone through the upregulation of auxin transporters (Vragović et al., 2015; Figure 2B). Besides, mutations in the auxin biosynthesis (*taa1*) or the efflux transport (*pin2*) genes revert the enlarged MZ size observed when *BRI1* is overexpressed only in the epidermis in the triple mutant *bri1 brl1 brl3* (Vragović et al., 2015). Furthermore, the MZ size of the triple *PILS* mutant (*pils235*) is more whereas the OE of *PILS5* is less inhibited to BR treatment than WT plants (Sun et al., 2010).

ABA downregulates the levels of auxin response in the PD (He et al., 2012; Figure 2B). Also, *ARF2* positively regulates MZ size in response to ABA, since its length is considerably reduced in the *arf2-101* mutants treated with ABA (Figure 2B). Besides, *arf2-101 pin4-3* and *arf2-101 pin1-1* double mutants, have a larger MZ than the single mutant (*arf2-10*1), suggesting that *PIN1* and *PIN4* counteract the ABA effects in the MZ size of *arf2-101* (Promchuea et al., 2017; Figure 2B). ABA negatively regulates *PLT1* and *PLT2* expression through *ARF2*, and the OE of *PLT2* in *arf2-101* mutant enhances MZ size compared to either the single mutant or the OE line and this phenotype can be partially inhibited by ABA treatment (Promchuea et al., 2017).

During the early stages of meristem development, GA regulates the MZ size by suppressing CK signaling (Moubayidin et al., 2010). GA targets the degradation of the DELLA protein RGA that positively regulates the expression of *ARR1* and *SHY2* thus repressing CK signaling and inducing auxin responses in the MZ. Also, auxin negatively regulates the expression of *SHY2* that down regulates PINs expression and, therefore, auxin movement (Dello Ioio et al., 2008b; Moubayidin et al., 2010; Figure 2B). In addition, in the single *arr1-3* and in the double *arr1-3 arr12-1* CK signaling mutants, the promoter effect of GA or the inhibitor effect of PAC at 5 dag, is lost and the MZ size is as in control conditions (Moubayidin et al., 2010; Figure 2B). Furthermore, the induction of ARR1 protein translocation into the nucleus causes a reduction in root MZ size that can be reverted by GA treatment that depletes DELLAs from PR cells (Marín-de la Rosa et al., 2015). These data suggest that the interaction between type-B ARR, DELLA and auxin transport proteins are required to establish the MZ size.

#### Transition Domain Regulation by Hormones

**FIGURE 3:**
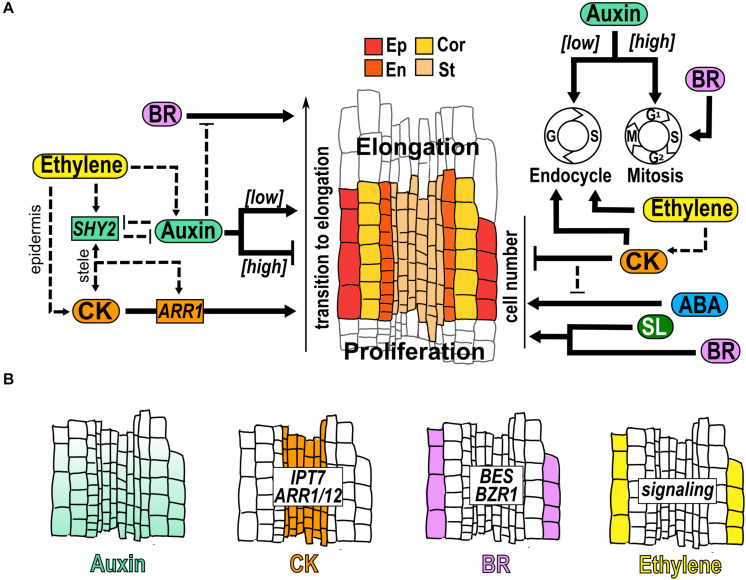
Hormone function in the transition domain. **(A)** Structure of the TD that is a region flanked by the dividing cells of the PD and the elongating cells in the EZ. The cells of different tissues are indicated: epidermis (Ep), cortex (Cor), endodermis (En) and stele (St); and hormonal regulation (black arrows) and crosstalk (dotted arrows) over the control of the transition to elongation, the TD cell number and the control of endocycle, conformed by Gap (G) and Synthesis (S); and Mitosis (M), conformed by Gap1 (G1), S, Gap2 (G2) and M is indicated. **(B)** Hormone accumulation (different colors), expression pattern of genes and hormonal pathways that participate in the TD.

The cell cycle of the transition domain (TD) cells is changing from cell division to endoreduplication, in which cells undergo several cycles of DNA replication with no cytokinesis (Verbelen et al., 2006; Bhosale et al., 2018; Salvi et al., 2020; Figure 3A). Moreover, these cells continue to grow at the same rate as in the PD but the frequency of division decreases resulting in the coexistence of cells that may still divide with others that undergo endoreduplication and are relatively larger than those in the PD (Ivanov and Dubrovsky, 2013). TD cells also have re-arrangements of the cell wall and cytoskeletal organization, high fluxes of ions, auxin and oxygen, and a high rate of vesicle recycling and vacuolization, to prepare for the fast elongation that takes place in the EZ (Verbelen et al., 2006; Baluška and Mancuso, 2013; Salvi et al., 2020). In addition, TD integrates external and internal signals, including hormonal responses, to determine cell fate and root growth (Baluška et al., 2010; Baluška and Mancuso, 2013; Kong et al., 2018). In this domain, the root cell elongation is controlled, among others, by α-expansins which are proteins that are activated by low apoplastic pH and allow the loosening of the cell wall and turgor−driven cell expansion (Cosgrove, 2005; Pacifici et al., 2018). Changes in the TD have been quantified as TD cell number, TD position regarding its distance from the QC or through the meristematic cell transition to elongation that can either reduced or increment the MZ (Dello Ioio et al., 2007, 2008b; Di Mambro et al., 2017; Pacifici et al., 2018).

It has been described that auxin, ABA and SL inhibit the transition from proliferation to elongation. An auxin minimum in the TD is necessary for the transition to elongation and perturbation of this minimum changes the TD position and the MZ size (Di Mambro et al., 2017; Kong et al., 2018; Figure 3B). In addition, lowering auxin levels reduces mitosis downregulating the expression of mitotic cell cycle genes and increasing the endocycle (Ishida et al., 2010; Figure 3A). As in the PD, PIN expression and distribution are also important in the TD to create the auxin reflux along the meristem (Blilou et al., 2005; Verbelen et al., 2006; Petrášek and Friml, 2009; Baluška et al., 2010; Salvi et al., 2020). Accordingly, different mutants defective in biosynthesis, transport or signaling of auxin, promotes the transition from mitotic to endocycle regulation and the TD appears proximally to the QC whereas increasing auxin levels, delays the onset to endocycling and elongation (Ishida et al., 2010; Figure 3A). Interestingly, the OE of *CYCA2;3* partially reverts the early entry into the endocycle and the short meristem induced by the auxin antagonist (2-(1H-Indol-3-yl)-4-oxo-4-phenyl-butyric acid; PEO-IAA) (Ishida et al., 2010). Also, the mutant of *GRETCHEN HAGEN 3.17* (*GH3.17; gh3.17–1*), a gene that encodes an IAA amino acid synthase conjugating auxin with amino acids to decrease the free auxin levels, shows an increment of free auxin and has a TD that appear distally with a delay in the onset to endocycling and cell differentiation (Di Mambro et al., 2017). Accordingly, the OE of *GH3.17* decreases auxin levels, and the position of the TD appears proximally with a premature transition to cell elongation compared to WT (Di Mambro et al., 2017).

ABA also suppresses the differentiation and positively regulates RAM size; application of ABA (0.5μM) increases the cell number in the TD resulting in a longer meristem. Accordingly, fluridone treatment, an inhibitor of ABA, reduces the TD cell number and the MZ size (Zhang et al., 2010; Takatsuka and Umeda, 2019; Figure 3A). Similarly, treatment with SL (2.5μM), increases the size of the TD and the cell number and decreases the cell length in the TD, compared to untreated plants, suggesting an effect of SL in the regulation of cell relative growth in the TD (Ruyter-Spira et al., 2011; Figure 3A).

CK, BR and ethylene are hormones that promote the transition from proliferation to elongation. CK synthesis and signaling genes such as *IPT7*, *ARR1* and *ARR12*, are expressed in the vascular tissue of the TD where CK regulates cell differentiation by antagonizing auxin signaling (Miyawaki et al., 2004; Dello Ioio et al., 2007; Salvi et al., 2020; Figure 3B). It has been described that the specific expression of *CKX1*, which catalyzes the degradation of CK, in the vascular tissue of the TD, results in an enlargement of the MZ and a delay in cell differentiation (Dello Ioio et al., 2007). Also, elevated CK levels change the position of the TD closer to the QC, leading to a shorter meristem, whilst low CK displaces the TD distally to the QC, generating a longer meristem (Dello Ioio et al., 2007). The treatment with CK reduces the number of TD cells and the CK signaling mutants *ahk3/4* and *arr2* have more TD cells than WT (Takatsuka et al., 2018; Takatsuka and Umeda, 2019; Figure 3A). Besides, CK stimulates the transition to endocycling through the positive and direct regulation of the anaphase promoting complex/cyclosome (APC/C) activator *CELL CYCLE SWITCH PROTEIN 52A* (*CCS52A1*) expression by ARR2 (Figure 3A; Takahashi et al., 2013).

Several expansins and H^+^-ATPases regulate cell growth in the TD and their expression depends partially on CK signaling (Pacifici et al., 2018). Besides, CK also participates in the reorganization of actin, which is involved in cell elongation that begins in the TD (Takatsuka et al., 2018). Moreover, the GoF of *PHB* (*phb-1d*), has a short meristem and the TD is closer to the QC compared to WT plants and, interestingly, the expression of *CKX1* in the TD is sufficient to restore the MZ size in *phb-1d/* + mutant indicating that *PHB* functions on TD are dependent on CK (Dello Ioio et al., 2012). Furthermore, *PHB* enhances the CK biosynthesis inducing the expression of *IPT7* in the pro-vascular cells of the meristem and it has been suggested that CK moves to the TD and promotes the transition of cells from the TD to the EZ (Bishopp et al., 2011; Dello Ioio et al., 2012).

In the TD, BR functions as a promoter of the transition from proliferation to elongation (Figure 3A). Accordingly, *bri1* mutant has a defective cell cycle activity and cell expansion, that leads to a significantly reduced TD, with less and shorter cells compared to WT (Hacham et al., 2011). Moreover, a similar phenotype is detected in the BR biosynthesis mutant *constitutive photomorphogenesis and dwarfism* (*cpd*), with few cells in the TD (Hacham et al., 2011; Figure 3A). In this domain, BR signaling is high in the two outermost cell types (epidermis and lateral root cap) but BR responsive genes are only high in the epidermis (Vragović et al., 2015). The concentration of the proteins encoded by the TFs *BZR1* and *BES* is highest in the epidermal cells of the TD and a gene construction that expresses the activated *BZR1* in the TD epidermis, rescues the reduction of MZ and EZ that occurs in *bri1-116* mutant (Chaiwanon and Wang, 2015; Figure 3B).

Ethylene treatment induces a rapid increase in endoreduplication and nuclear area in the TD compared to control conditions (Figure 3A) and this increment in endoreduplication is not present in the ethylene insensitive mutants (*etr1-1* and *ein2-50*) in response to ethylene (Street et al., 2015). It would be interesting to investigate if this regulation over endoreduplication affects the length and/or the number of TD cells.

In the TD an interplay between diverse hormones occurs; two of the most characterized that participate in the establishment and maintenance of this domain, are auxins and CK that are interlinked and act antagonistically (Dello Ioio et al., 2008a; Di Mambro et al., 2017; Kong et al., 2018; Salvi et al., 2020). This crosstalk relies on several mechanisms: ARR1 and ARR12 up-regulate the transcript accumulation of the auxin signaling repressor *IAA3*/*SHY2*, which is expressed in the vasculature of the TD (Figure 3A). SHY2 down regulates the transcript accumulation of several auxin efflux transporters affecting the accumulation and distribution of auxin in the TD (Dello Ioio et al., 2008b; Moubayidin et al., 2010; Takahashi et al., 2013; Figure 3A). In addition, auxin mediates the degradation of SHY2 that upregulates *IPT5* and *ARR1* transcript accumulation and CK biosynthesis in the vascular bundle of the TD (Dello Ioio et al., 2008b; Moubayidin et al., 2013) (Figure 3A).

Moreover, in the epidermal cells of the TD, BZR1 target genes tend to be induced by BR but repressed by auxin and the application of IAA causes a diminished nuclear accumulation of BZR1 and its subsequent localization in the cytoplasm, which is associated with low BR signaling (Chaiwanon and Wang, 2015; Figure 3A). Interestingly, ethylene also induces the expression of *SHY2* that is indispensable for the negative effect of ethylene on the MZ size (Street et al., 2015) (Figure 3A). Ethylene signaling response is detected in the epidermis of the TD (Figure 3B) where it regulates the pH of the apoplast via positive regulation of auxin biosynthesis and responsiveness in the root tip, showing the interdependence of ethylene and auxin to regulate root growth (Vaseva et al., 2018; Figure 3A). Furthermore, the CK-responsive construct (*pTCSn:GFP*) is also induced by ethylene predominately in the epidermal cells of the TD and it is abolished in the *etr1-1* mutant but not in *ein2-1* mutant, indicating that ethylene regulation of CK is via *ETR1* pathway but independently of the canonical *EIN2* pathway (Zdarska et al., 2019).

Finally, ABA treatment (0.5 and 10 μM) enhances the number of cells in the TD in WT plants but not in the *ahk3/4* and *arr2* mutants that show a similar number as in control conditions (Takatsuka and Umeda, 2019; Figure 3A).

### Hormone Function in the Elongation Zone

The cells in the elongation zone (EZ) have a rapid anisotropic growth accompanied by cell wall loosening, endoreduplication and changes in the organization of the microtubules (Dolan and Davies, 2004). In the EZ the cells do not divide and can increase their length three times in only 3 h (Verbelen et al., 2006; Salvi et al., 2020; Figure 1). Recently, it was discovered that in seedlings where the shoot is removed, the EZ is displaced toward the root tip, indicating that the aerial part of the plant can send signals to maintain root growth (Baskin et al., 2020). In addition, hormones regulate the expression and function of enzymes that participate in cell wall modifications in the EZ similarly to what happened in TD cells (Wu and Cosgrove, 2000; Somssich et al., 2016; Barbez et al., 2017; Dietrich, 2018). As we will discuss below, many hormones are involved in the regulation of cell elongation.

**FIGURE 4:**
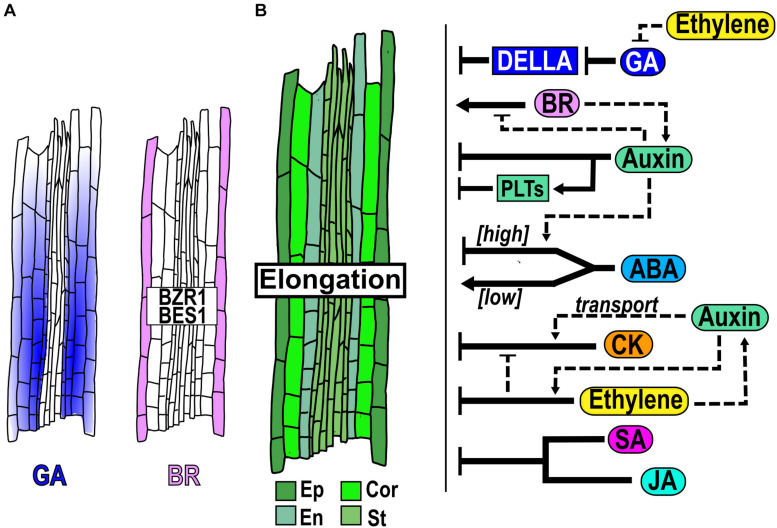
Hormone function in the elongation zone homeostasis. **(A)** Hormone accumulation and expression pattern of genes that participate in the EZ. **(B)** Structure of the EZ, with cells from different tissues: epidermis (Ep), cortex (Cor), endodermis (En) and stele (St) and hormonal regulation (black arrows) and crosstalk (dotted arrows) function in the EZ.

In the EZ, GA and BR are hormones that act as positive regulators of cell elongation. GA is synthesized mainly in the endodermal cells of the MZ but the bioactive GA is accumulated in the endodermal, cortical and epidermal cells of the EZ where it contributes to the regulation of cell elongation (Ubeda-Tomás et al., 2008; Shani et al., 2013; Rizza et al., 2017; Barker et al., 2020; Figure 4A); but GA-responses in the endodermis are sufficient to regulate cell expansion (Ubeda-Tomás et al., 2008; Rizza et al., 2019). Also, it has been proposed that the levels of GA in the EZ have a graded distribution due to dilution given the rapid cell growth, resulting in a subsequent increase in DELLAs concentration toward the end of the EZ that could function as a mechanism to terminate the elongation (Band et al., 2012). Lowering GA levels, with PAC, an inhibitor of GA synthesis, or blocking the GA response in endodermis, using a GA-insensitive mutant form of the DELLA protein GAI, provokes a reduction in cell elongation (Ubeda-Tomás et al., 2008, 2009; Band et al., 2012; Figure 4B).

Application of BR (100 nM) promotes cell elongation in the EZ and the BR signaling proteins *BZR1* and *BES* concentrate at high levels in the epidermal cells of this zone (Chaiwanon and Wang, 2015; Figure 4A). Besides, BR acts a positive regulator of cell elongation, since the LoF mutants *bri1-116* and the triple mutant *bri1 brl1 brl3* have a significantly shorter elongated cells, while the OE of *BRI1* and *bes1-D* have larger fully elongated cells than WT plants (González-García et al., 2011; Hacham et al., 2011; Kang et al., 2017; Figure 4B). Moreover, in the BR-deficient mutant, *dwf4*, the treatment with either low or high BR concentrations can rescue the size of the fully elongated cells (Chaiwanon and Wang, 2015). In addition, BZR1 is maintained at high levels in the nucleus in the EZ, promoting cell elongation via upregulation of genes induced by BR as cell-wall proteins and its expression in the epidermis rescues the size of the fully elongated cells of the *bri1-116* mutant phenotype (Chaiwanon and Wang, 2015), indicating that it is its main site of action (Figure 4A). Interestingly, *BRI1* expression promotes cell elongation of hair (trichoblasts) cells while inhibits in non-hair (atrichoblasts) cells (Hacham et al., 2011; Fridman et al., 2014; Vragović et al., 2015).

Several hormones such as CK, Ethylene, JA, SA and auxin, act as negative regulators of cell elongation. CK application reduces cell elongation (Figure 4B; Beemster and Baskin, 2000) and re−organized the cortical microtubules at the EZ from a transversal to an oblique disposition, which is associated with termination of cell expansion (Montesinos et al., 2020). Furthermore, the length of the completely elongated cells is larger in *arr1 arr12* double mutants than in WT plants (Street et al., 2016; Figure 4B). Similarly, application of either the ethylene precursor 1-aminocyclopropane-1-carboxylic acid (ACC), SA or JA inhibits cell elongation compared to WT plants (Rùžièka et al., 2007; Chen et al., 2011; Pasternak et al., 2019; Figure 4B). Moreover, the single or double LoF mutants of the JA negative regulator, *NOVEL INTERACTOR OF JAZZ* (*NINJA)* and of the gene that encodes the enzyme ALLENE OXIDASE SYNTHASE (AOS), have shorter fully elongated cells than WT plants (Acosta et al., 2013).

Auxin conjugation and auxin perception/signaling are important for apoplast acidification and promotion of cell elongation; the inducible expression of *GH3.6*, the triple mutant of the auxin receptors *TRANSPORT INHIBITOR RESPONSE1* (*TIR1*)*/Auxin-Binding F box* (*AFB*s) (*tir1afb2afb3*), the GoF of *bodenlos* (*iaa12/bdl*) mutant and the double mutant *arf10 arf16*, have a higher apoplastic pH with smaller EZ epidermal cells compared to WT (Barbez et al., 2017). Interestingly, the root cell elongation is also inhibited using a high IAA concentration (250nm) or in the OE of *YUC6* compared to control conditions, probably by a transitory alkalinization of the apoplast (Barbez et al., 2017; Figure 4B). Moreover, cells in the EZ have transversal microtubules that are more sensitive to auxin−driven reorientation of the microtubule network to longitudinal disposition than in other PR zones leading to a reduced cellular elongation rate (Montesinos et al., 2020). Additionally, many different PIN mutants have a reduction in the cell elongation size compared to WT and high levels of *PLT2* expression or PLT2 protein in the EZ inhibit cell elongation (Galinha et al., 2007; Mähönen et al., 2014) (Blilou et al., 2005).

In addition, the treatment with high levels of ABA (30μM) significantly reduces cell elongation, while low levels of ABA treatment (100 nM) increase it (Dietrich et al., 2017; Promchuea et al., 2017; Figure 4B). Furthermore, the *snrk2.2 snrk2.3* double mutant of the ABA signaling kinase is insensitive to the promotion of cell elongation at low (100 nM) ABA levels (Dietrich et al., 2017). Interestingly, the application of ABA (2μM) in the shoot promotes a significant increment in root cell elongation compared to control conditions. This might be mediated by long-distance transport of an output of the ABA signaling transduction pathway in the shoot, since this hormone does not diffuse into the roots (Xie et al., 2020).

There is an interplay between different hormones in the regulation of cell elongation. For instance, ABA treatment reduces the cell elongation of the *arf2-101* mutant, compared to WT plants, but does not alter the cell number (Promchuea et al., 2017). In the case of auxin and its interaction with ABA, the double mutants *arf2-101 pin1-1* and *arf2-101 pin4-3* have longer cells in the EZ than the *arf2-101* single mutant in response to ABA treatment (Promchuea et al., 2017; Figure 4B).

As described above, BR induce cell elongation, whilst auxin deficiency or high exogenous or endogenous concentrations, inhibits it (Barbez et al., 2017). Accordingly, auxin treatment induces cytoplasmic localization of BZR1 in the EZ reducing its nuclear accumulation that results in a decrease in BR signaling in this zone (Chaiwanon and Wang, 2015). While BR treatment (100 nM) promotes cell elongation, the co-treatment of BL with high auxin (5μM) enhances the inhibitory effect of auxin over cell elongation (Chaiwanon and Wang, 2015; Figure 4B).

Auxin interacts with CK and ethylene, which also function as negative regulators of cell elongation. The influx carrier AUX1 is required for the CK responses as the *aux1* mutant is insensible to CK inhibition of cell elongation compared to the WT (Street et al., 2016; Figure 4B). Additionally, the completely elongated cells of *aux1 arr12* and *aux1 arr1 arr12* mutants are also insensitive to CK inhibition compared to WT plants and control conditions, whereas the *arr12* single mutant has the same CK sensitivity as the WT, regarding the length of elongated cells (Chaiwanon and Wang, 2015). Also, high concentrations of ethylene inhibit epidermal cell elongation and this inhibition is dependent on the promotion of the basipetal auxin transport mediated by AUX1 and PIN2, as well as by the auxin biosynthesis in the EZ (Rùžièka et al., 2007; Swarup et al., 2007) (Figure 4B). Moreover, auxin function in the EZ makes cells more sensitive to ethylene; high auxin levels in the TD activate ethylene signaling in the EZ and represses cell elongation (Stepanova et al., 2007). Besides, ethylene effects on cell elongation are diminished in the *pin2*/*eir1-1, aux1-T* mutants and in the OE line of *PIN1*, indicating a key role of basipetal auxin transport in such responses (Rùžièka et al., 2007).

Interestingly, ethylene seems to act as a negative regulator of cell elongation by inhibiting GA uptake, accumulation and distribution in the endodermis of the EZ (Shani et al., 2013; Figure 4B). Furthermore, CK regulates the inhibition of cell elongation partially in an ethylene dependent pathway since the ethylene insensitive *ein2* mutant shows less reduction on cell elongation in the EZ in response to CK compared to WT (Street et al., 2016; Figure 4B).

### Differentiation Zone Regulation by Hormones

**FIGURE 5:**
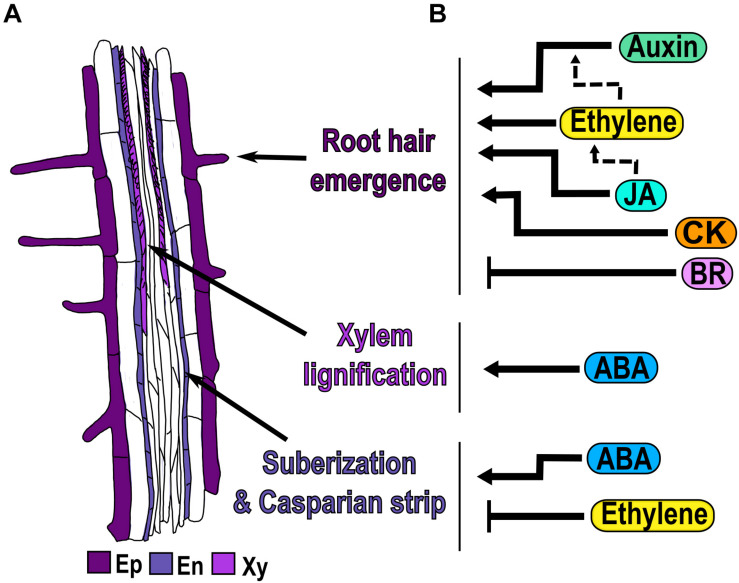
Hormonal regulation of cell differentiation. **(A)** Morphological markers of cell differentiation are the emergence of the first epidermal (Ep) root hair, the xylem (Xy) lignification, the suberization of endodermal (En) cells and the presence of the Casparian strip. **(B)** Hormone regulation (black arrows) and crosstalk (dotted arrows) in each of the three differentiation processes.

In the differentiation zone (DZ) the cells stop elongating and acquire the final characteristics of mature cells (Figure 1B). One of the morphological markers of the beginning of the DZ is the appearance of the first epidermal root hair (Dolan et al., 1993; Verbelen et al., 2006; Cajero Sánchez et al., 2018; Salvi et al., 2020; Figure 5A). Epidermal cells differentiate into trichoblasts and atrichoblasts, depending on their contact with the underlying cortical cells: cells contacting two cortical cells develop root hairs, whereas non-hair cells overlie just in one cortical cell (Schiefelbein et al., 2009; Shibata and Sugimoto, 2019). In Arabidopsis, these cells are organized in alternated files, with the hair cells files regularly separated by two non-hair cell files (Ishida et al., 2008). Other morphological characteristics of differentiated root cells are the formation of xylem secondary walls in the pro-vasculature, and the Casparian strip and suberization of endodermal cells (Dolan et al., 1993; Somssich et al., 2016; Cajero Sánchez et al., 2018; Figure 5A).

In this section we focus on the function of hormones in the emergence of root hairs, the lignification of xylem cells and the suberization of endodermal cells as developmental processes that take place in the DZ (Figure 5A). There are other excellent reviews that have information regarding root hair elongation (Carol and Dolan, 2002; Libault et al., 2010; Salazar-Henao et al., 2016; Vissenberg et al., 2020) and vascular patterning (Kondo et al., 2014; Smet and De Rybel, 2016; Vaughan-Hirsch et al., 2018; Ramachandran et al., 2020), processes that will not be addressed here.

Auxin, ethylene, CK and JA are hormones that act as positive regulators of root hair emergence in the DZ. The Arabidopsis hairless mutant *root hair defective 6 (rhd6)* can be rescued by the treatment with auxins, CK or ethylene that cause an increase in the frequency of root hairs (Masucci and Schiefelbein, 1996; Rahman et al., 2002; Zhang et al., 2016). Moreover, auxin is required for the initiation of root hairs and consequently, the GoF mutant *iaa17/axr3-1*, defective in auxin signaling, has no root hairs (Masucci and Schiefelbein, 1996; Knox et al., 2003; Lee and Cho, 2013; Shibata and Sugimoto, 2019; Figure 5B). In addition, in the recessive *aux1-7* mutant of an auxin influx transporter, only 30% of the epidermal cells develop root hairs, compared to 40% in WT; accordingly, the treatment with either NAA or the chromosaponin I (CSI), a compound that improves auxin uptake, increments the percentage of root hair-bearing cells in *aux1-7* (Rahman et al., 2002). In the null allele of *AUX1*, *aux1-22*, CSI application does not improve root hair formation, showing that CSI needs AUX1 to regulate root hair density (Rahman et al., 2002).

Ethylene application induces ectopic hair formation in atrichoblast cells (Cao et al., 1999; Dolan, 2001; Le et al., 2001; Zhang et al., 2016; Figure 5B). Consequently, inhibitors of ethylene biosynthesis (AVG) and perception (Ag^+^), block root hair formation in all cells (Tanimoto et al., 1995; Masucci and Schiefelbein, 1996; Figure 5B). Likewise, the *ethylene overproducer 3* (*eto3)* mutant, that produces elevated levels of ethylene, and the constitutively ethylene-responsive mutant *ctr1* both develop hairs in the epidermal cells in the position of atrichoblasts (Masucci and Schiefelbein, 1996; Cao et al., 1999). Furthermore, it has been described that ethylene promotes the initiation of root hairs via the interaction of ethylene signaling EIN3/EIL1, with the proteins encoded by the genes involved in root hair initiation and growth like *RHD6* and its respective paralog: *RHD6-LIKE (RSL1)* (Feng et al., 2017). In the *rhd6 rsl1* double mutant, with a hairless phenotype, the ACC treatment produces bulges that are characteristic of root hair initiation, and the quadruple mutant *ein3 eil1 rhd6 rsl1* do not produce these bulges even with ACC treatment (Feng et al., 2017). Auxin and ethylene seem to independently regulate root hair density as NAA application to *ein2-1* increases the frequency of root hairs (Rahman et al., 2002), whereas the root hairs density decreases with the auxin inhibitor 1-NOA in this mutant. Furthermore, the double mutant *aux1-7 ein2* has a frequency of root hairs emergence lower than the WT; and the single mutants with NAA treatment completely restores the root hair phenotype to WT (Rahman et al., 2002; Figure 5B).

The application of JA increases root hair density, while perturbation in JA perception/signaling as in the *coi1-2* mutant, causes a decrease in root hair frequency (Zhu et al., 2006, 2011; Figure 5B). Interestingly, the JA-induced root hair development is inhibited by the co-treatment with the ethylene inhibitors, AVG or Ag +, as well as in the *etr1-1* mutant, showing that these two hormones act in the same pathway regulating root hair density (Zhu et al., 2006, 2011; Figure 5B).

Conversely, BR is a hormone that acts as a negative regulator of root hair emergence, since the application of BR reduces the formation of root hair in trichoblasts, whereas the treatment with the BR inhibitor, brassinazole (Brz), leads to the formation of root hairs in atrichoblasts cells (Cheng et al., 2014; Figure 5B). In mutants related to BR synthesis: *det2-1*, *cpd*, a cytochrome P450 enzyme, and in the LoF of *BRI1* (*bri1-116*), the hair density is higher due to the presence of more hair files, suggesting that atrichoblast files change their fate to trichoblast. Therefore, plants with an increased BR signaling, as the OE of *BRI1* and the triple mutant of *BIN2* and their paralogs, (*bin2-3 bil1 bil2*), have fewer root hairs than WT, because many trichoblasts cells do not develop root hairs (Cheng et al., 2014).

ABA is a hormone that acts as a positive regulator of xylem differentiation and suberization of endodermal cells in the DZ. Mutants with reduced ABA levels (*aba2-1* and *aba3-1)* and plants treated with fluridone, have discontinuous or absent xylem strands compared to WT and this phenotype is reversed by ABA treatment (Ramachandran et al., 2018; Figure 5B). ABA is also involved in the lignification of the Casparian strip and suberization of endodermal cells, which are characteristics of differentiated endodermal cells. In response to ABA, the Casparian strip appears closer to the end of the TD compared to control conditions, showing that ABA promotes early endodermal differentiation (Bloch et al., 2019; Figure 5B). Moreover, suberin biosynthesis in endodermal cells is induced by ABA treatment, through the enhanced expression of the *GLYCEROL-3-PHOSPHATE ACYL-TRANSFERASE 5* (*GPAT5)*, a suberin biosynthesis enzyme (Barberon et al., 2016). Accordingly, mutants of ABA biosynthesis and response (*aba2*, *aba insensitive 3 [abi3], abi4* and *abi5*) have a delay in suberin deposition that is formed in a discontinuous pattern (Barberon et al., 2016; Figure 5B).

Ethylene is a hormone that acts as a negative regulator in suberization of endodermal cells in the DZ. Contrary to the effect of ethylene as a promoter of root hair emergence; the application of ACC reduces the suberin accumulation in newly differentiated endodermal cells via *GPAT5* (Barberon et al., 2016; Figure 5B). Interestingly, ACC also triggers the degradation of pre-existing suberin in already differentiated endodermal cells creating a patchy suberin pattern in the endodermis compared to control conditions (Barberon et al., 2016). Accordingly, mutants defective in the ethylene-signaling pathway, *ein3* and *etr1*, have enhanced suberization, while *ctr1* mutant, with constitutive ethylene response, has less suberization compared to WT (Barberon et al., 2016; Figure 5B).

### Hormonal Regulation of Columella Development

**FIGURE 6:**
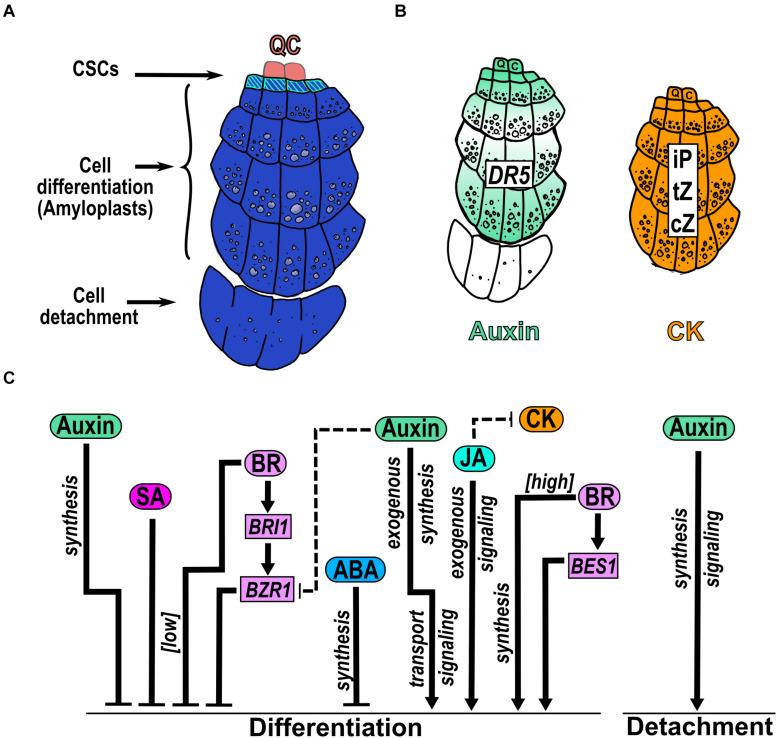
Hormonal regulation of CSC differentiation and COL detachment. **(A)** The columella (COL) is below the QC and is arranged in cell files of increasing size. The columella stem cells (CSCs) remain undifferentiated and originate COL differentiated cells that accumulate starch granules in amyloplasts. The last COL cell file is sloughed off from the root. **(B)** Presence of hormones in COL reported by the auxin response (DR5) and measurement of different CK types. **(C)** Hormone function over CSCs differentiation and COL sloughing. The comments that are nearby arrows indicate if the hormone was applied exogenously under [high] or [low] concentrations or if the regulation depends on genes that participate in hormone synthesis, transport or signaling. Dotted arrows indicate the hormonal crosstalk.

The columella (COL) and the lateral root cap (LRC) comprise the root cap that protects the epidermis and the SCN in the MZ. The root cap acts as an integrator of environmental information that is then transmitted to other root cells and provides lubrication that facilitates root tip penetration to the soil by secreting polysaccharide-based mucilage (Wen et al., 2007; Kumpf and Nowack, 2015). COL cells are localized at the root tip below the SCN and are organized in cell files of increasing size departing from the columella stem cells (CSCs) (Figure 6A). Columella differentiated cells contain amyloplasts (plastids with starch) that participate in root gravitropism and are excellent markers of columella cell differentiation (Dolan et al., 1993; Kumpf and Nowack, 2015; Figures 1, 6A). Moreover, COL has a controlled cell file number homeostasis with a high cell turnover that maintains a constant cell file number showing the tight coupling of cell proliferation, differentiation and detachment (Kumpf and Nowack, 2015; Kumar and Iyer-Pascuzzi, 2020). The regulation of cell proliferation and cell differentiation of the COL is maintained by a well-documented signaling gene regulatory network centered in the activity of *WUSCHEL-RELATED HOMEOBOX 5* (*WOX5*), a homeodomain TF expressed in the QC cells. WOX5 moves from the QC cells to the CSCs, where it negatively regulates *CYCLING DOF FACTOR 4* (*CDF4)* expression, that promotes CSCs differentiation (Pi et al., 2015). Thus, WOX5 constitutes a mobile signal emanating from the QC cells, to maintain the columella initials in an undifferentiated state. Besides, *CLAVATA3/EMBRYO-SURROUNDING REGION 40 (CLE40*), that modulates CSCs differentiation, also acts as a negatively regulator of *WOX5* expression via the receptor-like kinase *ARABIDOPSIS CRINKLY 4 (ACR4*) (Stahl et al., 2009; Drisch and Stahl, 2015; Richards et al., 2015). In addition, PLTs positively regulate *WOX5* expression to maintain the CSCs fate (Shimotohno et al., 2018). Interestingly, in this regulatory network WOX5 excludes the NAC [NO APICAL MERISTEM (NAM), ATAF, and CUP SHAPED COTYLEDON (CUC)] TF SOMBRERO (SMB), from the CSCs to promote differentiation (Bennett et al., 2014.). Moreover, it has been reported that other members of the NAC family of TFs like *FEZ* inhibits differentiation while *BEARSKIN1 (BRN1)* and *BRN2*, participate in COL cell detachment (Willemsen et al., 2008; Bennett et al., 2010; Kumar and Iyer-Pascuzzi, 2020). This latter process requires cellulases (Del Campillo et al., 2004; Figure 6A) and when cell division is repressed by either hydroxyurea or aphidicolin treatments, the cell detachment process stops and the number of differentiated columella cell files remains constant (Dubreuil et al., 2018). Also, removing the root cap, using a root cap specific promoter that drives the expression of the diphtheria toxin that kills cells, shows abnormal root growth with short meristem, fewer files of differentiated columella cells and abnormal phenotypes in the remaining columella files, showing the importance of this tissue in overall root development (Tsugeki and Fedoroff, 1999).

The participation of auxin, CK, ABA, BR, JA and SA, have been documented in the CSCs differentiation being auxin and CK two hormones that are highly accumulated in COL (Antoniadi et al., 2015; Dubreuil et al., 2018). Auxin homeostasis in the columella is affected by both long-range auxin transport and from local root synthesis (Dombrecht et al., 2007). The polar transport of auxin in the root generates a graded auxin distribution in the MZ which creates a maximum of auxin concentration and response in the QC cells followed by the CSCs and the outermost columella layer, separated by cell files with lower auxin responses (Petersson et al., 2009; De Rybel et al., 2012; Dubreuil et al., 2018; Kumar and Iyer-Pascuzzi, 2020; Figure 6B). The expression of tryptophan monooxygenase (*iaaM*), an *Agrobacterium tumefaciens* auxin biosynthesis gene, in the outermost COL cell layers, shows an increased number of attached COL cells (Dubreuil et al., 2018), indicating a role of auxin in the cell-cell separation process. Regarding CK, its nucleotides; ribosides, as isopentenyladenine (iP), cis-zeatin (cZ) and trans-zeatin (tZ); and its conjugates are found at high concentrations in both the CSCs and the differentiated COL cells. Accordingly, several genes that participate in auxin and CK homeostasis are expressed in COL (De Rybel et al., 2012; Antoniadi et al., 2015; Figure 3B).

The application of the synthetic auxin, 1-naphthaleneacetic acid (NAA), promotes differentiation of CSCs (Ding and Friml, 2010; Figure 6B). Besides, auxin transport, biosynthesis and signaling are required to promote CSCs differentiation; as the quadruple *PIN* mutant (*pin1 pin3 pin4 pin7*) completely aborted columella growth (Dubreuil et al., 2018); and the single mutants of *pin4, pin3* and *pin7*, the double mutant *arf10arf16*, mutants defective in tryptophan-dependent auxin biosynthesis genes as *YUCCA* (*yuc*) and *TAA1* (*wei8*), and mutants defective in auxin signaling as *INDOLE-3-ACETIC ACID INDUCIBLE 17*/*AUXIN RESISTANT 3* (*iaa17*/*axr3*) have multiple layers of CSCs (Friml et al., 2002; Ding and Friml, 2010). Likewise, the double mutant *arf10 arf16* shows detachment defects of the outermost COL cells (Wang et al., 2005; Figure 6B). Intriguingly, the LoF mutants of another allele of *TAA1* (*taa1^*ckrc*1–1^*) and the auxin biosynthesis anthranilate synthase double mutant (*asa1 asb1*), have a reduced COL size attributed to both less cell division and cell detachment (Dubreuil et al., 2018), contrary to what was reported by Ding and Friml (2010). As Dubreuil et al. (2018) did not analyze CSCs number and/or the presence of amyloplast in COL differentiated cells and only measure COL size, it is difficult to compare the two studies and it would be interesting to perform the same experimental procedures to analyze the auxin levels in each *TAA1* allele, to understand the auxin roles in CSCs differentiation.

Additionally, auxin participates in the regulatory network of COL promoting cell differentiation mediated by the repression of *WOX5* by ARF10 and ARF16 that restricts its expression to the QC cells (Ding and Friml, 2010). Moreover, auxin also promotes *WOX5* expression, through PLT to maintain the CSC fate (Shimotohno et al., 2018). At the same time, WOX5 regulates positively the expression of the auxin biosynthetic gene *YUC1* (Tian et al., 2014). The OE of *WOX5* increases auxin levels in PR and generates extra layers of CSCs, contrary to the phenotype of NAA application (Sarkar et al., 2007; Tian et al., 2014). Besides, in the LoF mutant of *WOX5* the CSCs are differentiated, opposite to the phenotype of auxin mutants mentioned above (Sarkar et al., 2007). Therefore, there is a multistability in the responses of WOX5 to auxin, such that the effect of auxin may depend on the cellular context in COL development (García-Gómez et al., 2017).

To our knowledge, the concentration profile of other hormones has not been quantified in COL, but their participation has been studied either by their application or by using LoF or GoF mutants.

JA promotes premature differentiation of CSCs and, consequently, an increasing number of COL cell layers with amyloplasts (Figure 6C). Curiously, the CSCs marker *J2341* is found in several columella cell layers under this condition indicating an increase in the cell files with CSCs identity (Chen et al., 2011). Besides, JA perception/signaling is necessary to induce CSCs differentiation as the mutants of *MYC2* and *COI1* do not differentiate under JA application. Accordingly, the RNA interference lines of a negative regulator of JA signaling (*JAZ10*) shows more CSCs differentiation compared to WT plants (Chen et al., 2011). Interestingly, exogenous application of JA reduces the signal of the CK-responsive marker *ARR5:GFP* in COL (Jang et al., 2019; Vázquez-Chimalhua et al., 2019) suggesting an antagonistic crosstalk between JA and CK (Figure 6C).

Otherwise, ABA and SA function as negative regulators of CSCs differentiation. Inhibition of ABA biosynthesis by fluridone, promotes CSCs differentiation and ABA induces the expression of *WOX5* and *PLT2* that are genes involved in COL development (Zhang et al., 2010; Figure 6C). Also, addition of low SA levels (30μM) produces an enlargement of CSCs area with two to four disorganized extra CSCs tiers whereas at higher SA levels (150 μM), COL architecture changes to bigger cells that lack starch granules (Pasternak et al., 2019; Figure 6C).

BR is a hormone that regulates CSCs differentiation in a dose-dependent manner; at low concentrations, BR inhibits CSCs differentiation and reduces the number of COL cells layers; in contrast, higher concentrations of BR have the opposite effects (González-García et al., 2011; Lee et al., 2015; Figure 6C). Also, BR signaling mediated by the positive regulators *BES1* and *BZR1*, have opposite functions on CSC differentiation i.e., the GoF mutant of *BES1* (*bes1-D*) enhances CSCs differentiation, while the GoF mutant (*bzr1-D*) enhances the number of CSCs layers with less COL differentiate cell layers (Lee et al., 2015; Figure 6C). Moreover, the mutant of the receptor *BRI1* (*bri1-116*) shows the same phenotype as *bes1-D* while the LoF of *de*-*etiolated 1* (*det1-2*), which participates in BR biosynthesis, has the same *bzr1-D* phenotype (Lee et al., 2015; Figure 6C). These results show that higher BR concentrations as well as the BES-mediated perception/signaling pathway stimulates CSC differentiation while lower BR concentrations and the BZR-mediated signaling pathway inhibits it (Lee et al., 2015; Figure 6C). Interestingly, it has been reported that BES acts as a transcriptional activator (Yin et al., 2005) whereas BZR1 as a repressor (He et al., 2005) so it would be very interesting to determine cell type specific BR target genes in different hormone concentrations and signaling and biosynthesis mutants. In addition, the lack of CSCs layers of *bri1-116* could be rescued by the OE of the D-Type Cyclin *CYCD3;1*, which promotes supernumerary layers of CSCs, in the double mutant (*bri1-116 CYCD3;1OE).* Accordingly, BR treatment in the OE of *CYCD3;1* plant, enhances the CSCs differentiation suggesting that BR regulates this phenotype through cell cycle regulation (González-García et al., 2011; Lee et al., 2015).

Besides, BR and auxin act antagonistically in columella development. The *bzr1-D* mutant phenotype, with additional layers of CSCs, is reverted into plants with more CSCs differentiation using NAA treatments (Lee et al., 2015; Figure 6C). Moreover, the inhibition of local auxin biosynthesis by L-kynurenine in the QC increases the nuclear accumulation and TF activity of *BZR1-YFP* in CSCs suggesting that the BZR1 pattern and nuclear accumulation is regulated by the auxin gradient (Chaiwanon and Wang, 2015). Interestingly, the nuclear accumulation of BZR1 causes extra divisions in the QC (Lee et al., 2015) that could affect the cell file number of CSCs.

The hormone participation in the regulation of the NAC TFs and their involvement in the regulatory network for COL development has not been addressed, different hormones might be regulating the expression of the NACs as their phenotypes in COL development are very similar. For instance, *fez* mutant promotes CSCs differentiation (Willemsen et al., 2008; Hong et al., 2015) and this phenotype is detected in the GoF mutant of *BES1*, in high BR, auxin or JA treatment and in auxin mutants. Contrary, the *smb-3* reduces CSCs differentiation as it is observed in the treatment with low BR and SA, as well as in BR signaling mutants and in auxin and ABA synthesis mutants (Willemsen et al., 2008; Ding and Friml, 2010; Chen et al., 2011; Lee et al., 2015). Furthermore, in the *brn1-1 brn2-1* double mutant, the COL cells fail to detach as is observed in auxin signaling mutants (Wang et al., 2005).

In brief, JA promotes COL cell differentiation while ABA and SA delay it. Interestingly, SA has a dose-dependent regulation over COL architecture that is not observed in any other zone or domain of the root. Furthermore, BR acts in a dose-dependent manner: at low concentrations repress, while at high concentration promotes, CSCs differentiation (Figure 6C). Besides, auxin can either promote or inhibit CSCs differentiation. Finally, auxin is the only hormone that participates in cell detachment, proliferation and differentiation (Figure 6C).

### Lateral Root Cap Regulation by Hormones

The Lateral Root Cap (LRC) is the cell file that surrounds and protects the external part of the MZ of the root and is derived from the LRC/epidermis stem cells (Figure 1; Dolan et al., 1993; Kumpf and Nowack, 2015). The cells of the LRC divide anticlinally and eventually die as part of a genetically controlled cell death program (PCD) upon reaching the EZ (Fendrych et al., 2014; Kumpf and Nowack, 2015; Huysmans et al., 2018). Hormone function over LRC development has been studied through changes in the MZ size (Xuan et al., 2016; Di Mambro et al., 2019).

**FIGURE 7:**
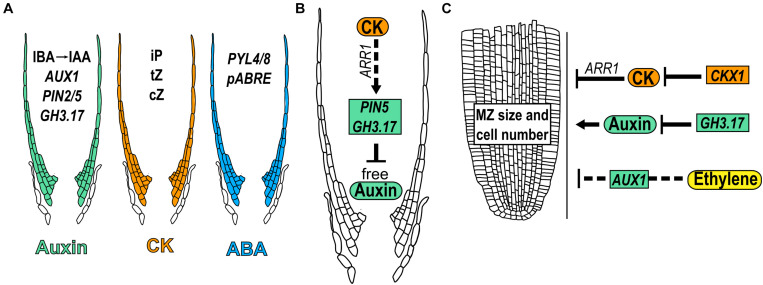
Hormone function in meristem size through lateral root cap. **(A)** Presence of different hormone forms and expression of related genes that participate in lateral root cap growth. **(B)** Regulatory crosstalk between auxin and CK in the lateral root cap development. **(C)** Hormonal regulations in LRC that modulate meristem size. Dotted arrows indicate the hormonal crosstalk.

CK and auxin-related proteins that participate in their transport or signaling, have been detected in the LRC cells (Antoniadi et al., 2015; Di Mambro et al., 2019). In the case of auxins, the expression in the LRC of the auxin influx transporter, *AUX1* and the efflux transporter *PIN2*, are required to mobilize the auxins from the MZ till the beginning of the EZ (Swarup et al., 2001; Feraru and Friml, 2008; Figure 7A). The endoplasmic reticulum auxin transporter *PIN5* is expressed in the LRC and transports auxin from the cytoplasm into the lumen of the endoplasmic reticulum; its OE results in decreased levels of free auxin in the LRC (Mravec et al., 2009; Di Mambro et al., 2019). In addition, *GH3.17* is expressed in the LRC and in EZ epidermal cells where it reduces free auxin levels (Di Mambro et al., 2019; Figure 7A). Additionally, outer LRC cells constitute a local source of auxin produced from indole-3-butyric acid (IBA) (Xuan et al., 2015). Regarding CK, the most active (iP, cZ, and tZ) precursors and conjugated forms, are accumulated at very high levels in LRC cells suggesting its restricted metabolism in these cells (Antoniadi et al., 2015; Figure 7A). Interestingly, *PIN5* and *GH3.17* are positively regulated by CK in the LRC through *ARR1* suggesting that auxins concentration and distribution in LRC cells are dynamically controlled by CK (Di Mambro et al., 2019; Figure 7B).

The expression of the ABA receptors *PYRABACTIN RESISTANCE–LIKE 4* (*PYL4)*, *PYL8* as well as the ABA-responsive element reporter (*pABRE_A:GFP)* are expressed in the LRC in early stages of root growth (Antoni et al., 2013; Belda-Palazon et al., 2018; Wein et al., 2020; Figure 7A).

Furthermore, hormonal concentrations in the LRC affect the development of other parts of the root; the presence of auxin and CK in the LRC affects the MZ size. In addition, the expression of *GH3.17* using the inducible LRC specific line (J2632), reduces meristem size while the mutant *gh3.17-1* displays an enlarged meristem; interestingly, the complementation of the *gh3.17-1* mutant, with the specific expression of *GH3.17* in the LRC, rescued this phenotype (Di Mambro et al., 2019; Figure 7C). Contrary to auxin, reducing the CK levels in LRC by using the J2632 reporter line to drive the expression of the gene that participates in its catabolism, *CKX1*, increases MZ size; accordingly, *ARR1* induction (with a high CK response) in the LRC (J2632 line), decreases the MZ size (Di Mambro et al., 2019; Figure 7C). Furthermore, CK regulates the expression of *GH3.17* in the LRC through *ARR1* (Di Mambro et al., 2019). These data suggest that auxin and CK levels in the LRC are determinant for the control of MZ size and that the effect of auxin in LRC is partially controlled by CK.

Furthermore, the PR growth regulated by ethylene depends on the transport of auxin via the LRC, as the expression of *AUX1* in LRC cells using the line *M0013* ≫*AUX1*, is enough to re-establish the ethylene inhibition of root growth in *aux1* mutant (Swarup et al., 2007; Figure 7C).

In summary, the LRC is a structure that protects but also regulates root MZ size and PR development. High levels of CK in the LRC inhibits while low levels of auxin promote the MZ size and the downregulation of auxin by CK in the LRC, partially controls its effect as a promoter of root MZ size.

## Conclusion

**FIGURE 8:**
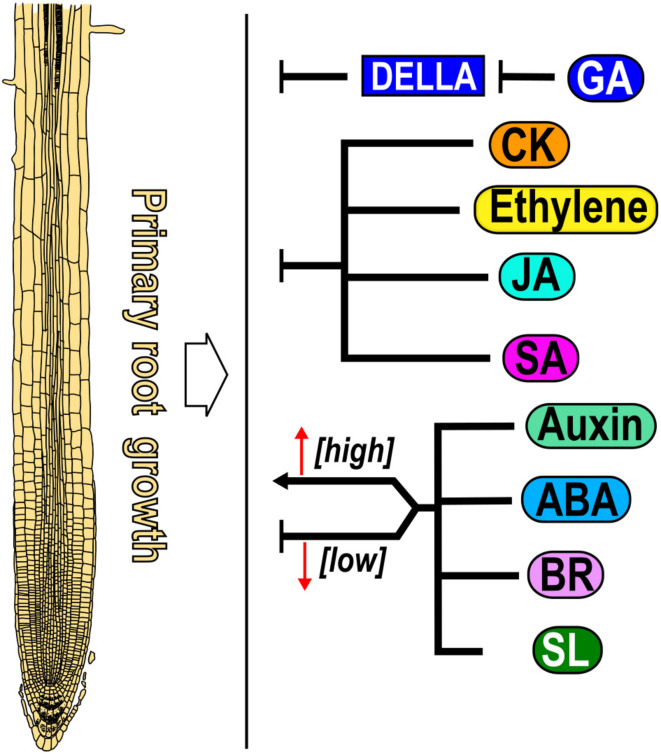
Effect of nine hormones in primary root growth. Gibberellic acid (GA) is a positive regulator, whereas auxin, abscisic acid (ABA), brassinosteroid (BR) and strigolactone (SL) act both as promoters and repressors of primary root growth in a dose-dependent manner. Cytokinin (CK), ethylene, jasmonic acid (JA) and salicylic acid (SA) are negative regulators of PR growth (black arrows). For all Figures, directed arrows represent positive whereas blunt arrows indicate negative regulation over root growth.

The balance between cell proliferation, elongation and differentiation is crucial for root growth and is dynamically adjusted depending on external and internal factors such as phytohormones. This review describes how nine hormones alone or in crosstalk, determine the different activities found in zones and domains of the Arabidopsis PR as despite the spatio-temporal roles over PR growth of the different hormones, it is difficult to understand how their synthesis, distribution and sensing accounts for their function in cell and tissue patterning. The PR growth is regulated in a dose-dependent manner; where low concentrations promote it and high concentrations inhibit it by auxin, ABA, BR, and SL (Müssig et al., 2003; Ruyter-Spira et al., 2011; Li et al., 2017; Waidmann et al., 2020; Figure 8), CK, ethylene, JA and SA act as negative regulators of PR growth (Medford et al., 1989; Le et al., 2001; Pasternak et al., 2005; Rùžièka et al., 2007; Kuderová et al., 2008; Chen et al., 2011; Figure 8) and GA is the only hormone that functions as a positive regulator of PR growth as different GA loss-of-function mutants and plants treated with an inhibitor of GA synthesis (Paclobutrazol, PAC) have shorter roots than WT (Fu and Harberd, 2003; Griffiths et al., 2006; Ubeda-Tomás et al., 2008, 2009; Figure 8).

PR growth is determined by the balance between cell proliferation and elongation. Accordingly, hormones that promote (GA) or inhibit (CK, ethylene, JA and SA) these two processes produce longer or shorter PRs, respectively. It is also possible that the readout of a hormone is not so straight such that one of these two cell processes compensate for the other, or that a gradient of concentration determines the root patterning as with auxin where it is found a maximum of concentration in the SCN followed by intermediate levels in the MZ and low levels in the EZ. Moreover, low levels of BR are important for MZ maintenance while BR signaling in the epidermal cells of the TD is necessary to promote cell elongation. Interestingly, hormone function also could be determined in each domain, zone or organ by changing the threshold of concentration where cells and tissues respond. Low concentrations of auxin, ABA, SL and BR promote while high concentration inhibit PR growth and the same occurs for ABA and BR in PD activity, for auxin in the TD to induce the transition to elongation and for ABA in cell elongation. Curiously, BR dose-dependent participation in CSCs, functions in opposite ways, low concentrations and the BZR1-mediated signaling pathway inhibit, while high concentration as well as the activation of the BES1 signaling pathway promotes CSCs differentiation. In addition, the BR effect over cell proliferation depends on the particular cell type where it is active; in the epidermis of the MZ, BR signaling is necessary to induce the MZ size, whilst in the stele it promotes cell differentiation. Furthermore, the MZ length of the single mutant of *bri1* and the triple mutant of *bri1 brl1 brl3* are bigger than WT if *BRI1* is expressed in the epidermal non-hair-cells. All these data suggest the importance not only of BR dosage but also the cell type where it is active to control the MZ size.

Moreover, we would like to highlight some important hormone crosstalks that are relevant to the activity and growth of the different zones and domains. A well-known crosstalk is the one where CK interacts antagonistically with auxin in the transition from the MZ to the TD and to the EZ where higher CK levels and lower auxin levels are necessary for endoreduplication. Interestingly, auxin and CK negatively regulate cell elongation and positively regulate root hair emergence suggesting different interactions between these two hormones in zones and domains. Also, ethylene inhibits the MZ size, promotes the early transition to EZ and suppresses cell elongation by both enhancing auxin biosynthesis and transport to the EZ, and inhibiting GA accumulation in the endodermis of the EZ.

It would be interesting to analyze how roots dynamically integrate all hormonal signals between the different zones and domains to alter the molecular processes underneath the changes in the ratio between cell proliferation, elongation and differentiation, to fully understand the PR development.

## Author Contributions

EZ-M, BL-R, and AG-A conceived and wrote the review. BG-P, MP, MG-G, and EÁ-B helped in writing and editing the review. All authors have read and agreed to the published version of the manuscript.

## Conflict of Interest

The authors declare that the research was conducted in the absence of any commercial or financial relationships that could be construed as a potential conflict of interest.
