# Supplementary material for: Integrative Roles of Phytohormones on Cell Proliferation, Elongation and Differentiation in the Arabidopsis thaliana Primary Root
Source: Front Plant Sci. 2021 Apr 26;12:659155. doi: 10.3389/fpls.2021.659155 (PMC8107238; doi:10.3389/fpls.2021.659155)
Supplement: Supplementary file 1 [file Data_Sheet_1.PDF]

Supplementary Material

1 Supplementary Figure

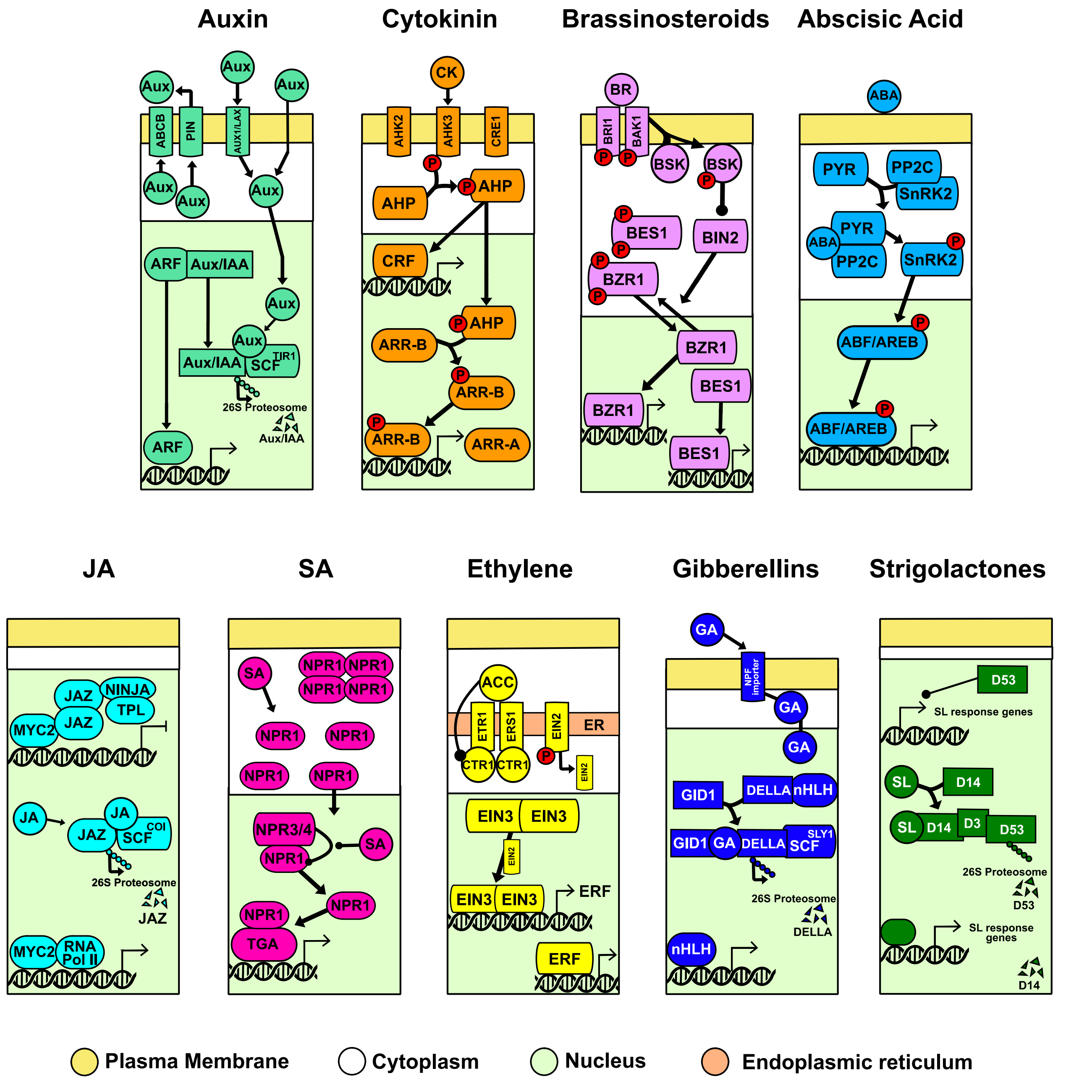

Figure S1. Basics of hormonal signal transduction. Auxin is a mobile hormone that can be imported from the apoplast passively and also by influx transporters of the AUXIN1/LIKE-AUX1 (AUX/LAX) gene family (Santner et al., 2009). The export of auxin is mediated by the auxin efflux transporters PIN-FORMED (PIN) and ARABIDOPSIS THALIANA ATP-BINDING CASSETTE B (ABCB). The auxin signaling pathway is elicited upon the binding of auxin to the F-box family

proteins TIR1/AFBs (TRANSPORT INHIBITOR RESPONSE 1/Auxin-Binding F box) and members of the Aux/IAA family of transcriptional repressors, promoting the ubiquitination of Aux/IAAs by the SCF complex. Otherwise, the AUX/IAA transcriptional repressors form dimers with AUXIN RESPONSE FACTORS (ARFs) inhibiting their activity as transcription factors. The auxin-induced degradation of Aux/IAA releases the ARFs that can in turn bind the auxin response elements and regulate the expression of auxin responsive genes. Cytokinin is perceived by transmembrane receptors with kinase function: ARABIDOPSIS HISTIDINE-KINASE RECEPTORS2 (AHK2), AHK3 and CRE1/AHK4/WOL that upon CK-binding auto-phosphorylate (Santner et al., 2009). The signal is transfered to the nucleus via a multi phosphorelay system mediated by proteins of the ARABIDOPSIS HISTIDINE-CONTAINING PHOSPHOTRANSFER PROTEIN (AHP) family. These proteins shuttle between the cytoplasm and the nucleus, where they donate the phosphate to the type A and B Arabidopsis Response Regulators (A-type and B-type ARR). B-type ARRs (ARR1, 2, 10–14, 18–21) are positive regulators of CK signaling that transcriptionally regulate the accumulation of CK responsive genes, including the A-type ARRs (ARR3-9,15–17) that act as negative-feedback regulators of CK signaling, and cytokinin response factors (CRF), AP2/ERF transcription factors that also mediate CK responses. Brassinosteroids (BR) are sensed by the transmembrane receptors like BRASSINOSTEROID INSENSITIVE1 (BRI1) that when activated, disassociate from BKI1 (BRI1 KINASE INHIBITOR 1) and associates with BAK1 (BRI1 ASSOCIATED KINASE 1) (Planas-Riverola et al., 2019).The heterodimerization with BAK1 initiate an intracellular phosphorylation cascade, in which BRASSINOSTEROID-SIGNALING KINASE (BSK) participates, that results in the dephosphorylation of the transcription factors BRASSINAZOLE RESISTANT 1 (BZR1) and BRI1-EMS-SUPPRESSOR 1 (BES1). Unphosphorylated BZR1 and BZR2 translocate to the nucleus, where they regulate the expression of BR-responsive genes. When the BR signaling pathway is inactive, BRASSINOSTEROID INSENSITIVE 2 (BIN2), a GSK3-like kinase, phosphorylates BZR1 and BES1. In the presence of BR, BIN2 is inactivated by BRI1 SUPPRESSOR 1 (BSU1) and marked for degradation. Abscisic acid (ABA) signaling is elicited by the binding of ABA to the receptors of the PYR/PYL/RCAR family (Pyrabactin Resistance/Pyrabactin resistance–Like/Regulatory Components of ABA receptors), inactivating the negative regulators ABI1 and ABI2 (ABA-Insensitive1 and 2) that are protein phosphatases of type 2C (PP2Cs); their inactivation leads to the phosphorylation of Snf1-Related protein Kinases2s (SnRK2s) that, in turn, phosphorylate AREB/ABF-type bZIP transcription factors to activate ABA-responsive gene expression (Santner et al., 2009). Jasmonic acid (JA) is perceived by the receptor complex CORONA INSENSITIVE1 (COI1)-JASMONATE ZIM DOMAIN PROTEINS (JAZ) (COI1-JAZ), leading to the ubiquitination and degradation of the JAZ negative regulators. This releases many TFs, mostly of the bHLH family, like MYC2, that can now interact with the Mediator Complex to recruit the RNA-pol II to JA-responsive genes. Otherwise, JAZ proteins repress transcription by binding MYC2 and recruiting TOPLESS (TPL), TPL-Related (TPR), Novel Interactor of JAZZ (NINJA) and epigenetic modifier proteins to negatively regulate the expression of JA-responsive genes (Santner et al., 2009). Salicylic acid (SA) signaling promotes the dissociation of NONEXPRESSOR OF PATHOGENESIS-RELATED GENES 1 (NPR1, an ankyrin-repeat protein) oligomers found in the cytoplasm, such that the NPR1 monomers can translocate to the nucleus where they interact with bZIP TFs and bind to the TGA (TGACGTCA cis-element-binding) proteins and regulate gene expression to promote defense responses and systemic acquired resistance. NPR3 promotes the degradation of NPR1 in a SA-dependent manner, whereas NPR4 promotes it constitutively (Santner et al., 2009; Fu and Dong, 2013). Ethylene can be perceived by ETHYLENE RESPONSE 1 (ETR1), ETR2, ETHYLENE RESPONSE SENSOR 1 (ERS1), ERS2 or ETHYLENE INSENSITIVE 4 (EIN4) receptors that are localized at the plasma membrane of the endoplasmic reticulum. These receptors act as negative regulators of the signaling transduction pathway in the absence of ethylene, by binding to the serine/threonine kinase Constitutive Triple Response 1 (CTR1). Upon ethylene perception, the receptor complex dissociates, CTR1 is inactivated, and the positive regulator of ethylene responses, EIN2, is cleaved such that a processed C-terminal product is translocated to the nucleus where it stabilizes EIN3 and EIN3-like (EIL1) TFs that activate the expression of *ETHYLENE RESPONSE FACTOR* (*ERF*) TFs, that can then subsequently regulate the expression of ethylene-responsive genes (Santner et al., 2009). Gibberellic acid (GA) can be imported to cells via the NPF3 importer (Tal et al., 2016). GA perception takes place in the nucleus, and involves the formation of a protein complex with the nuclear receptor GIBBERELLIN INSENSITIVE DWARF 1 (GID1) and the DELLA proteins. The GID-GA-DELLA complex favors the interaction between DELLA and the E3 ubiquitin ligase SCF SLEEPY 1 (SLY1) complex, resulting in the ubiquitination and eventual degradation of the DELLa proteins. The absence of DELLAs results in the release of many transcription factors (e.g. of the HLH family) that can now regulate GA-responsive genes (Santner et al., 2009). Strigolactones (SL) bind to the receptors DWARF14 (D14), an alfa/beta hydrolase protein, eliciting the binding of the F-box protein D3/MAX2 and of the transcriptional repressor D53 (SUPPRESSOR OF MAX2 1-LIKE (SMXL) 6/7/8 (Marzec, 2016). This results in a conformational change that causes the degradation of SL and the ubiquitination of D53, which is eventually degraded. D53 negatively regulates the expression of SL-responsive genes, such that its SL-dependent degradation allows in their expression. Then, D14 is degraded.

The perception of strigolactone (SLs) depends on an alfa/beta hydrolase superfamily protein DWARF14 (D14) that functions as a receptor and as an enzyme enabling SLs signaling. After the perception of SLs by the binding pocket of DL14, the F-box protein: D3/MAX2 binds to D14 with its displaced C-terminal helix, leading to D14 in an inactive state. Afterwards, the transcriptional repressor D53 (SUPPRESSOR OF MAX2 1-LIKE (SMXL) 6/7/8 in Arabidopsis) binds to D3 that origins D3 to recover its dislodged helix, returning the catalytic activity of D14 in order to degradete the SL molecule. Subsequently, D53 is polyubiquitinated and degraded allowing the expression of SL signaling pathway genes. Finally D14 is degraded and D3 is reused.

Table S1. Recommended reviews with hormonal information about biosynthesis, perception and signal transduction pathways for auxin, CK, GA, BR, ABA, JA, ethylene, SA and SL.

Hormone	References
Auxin	(Teale et al., 2006; Zhao, 2012; Ljung, 2013; Leyser, 2018),
Cytokinin (CK)	(Hwang and Sakakibara, 2006; Werner and Schmülling, 2009; Kieber and Schaller, 2014, 2018)
Gibberellic acid (GA)	(Schwechheimer, 2012; Davière and Achard, 2013; Gupta and Chakrabarty, 2013; Rizza and Jones, 2019)
Brassinosteroid (BR)	(Chung and Choe, 2013; Vukašinović and Russinova, 2018; Nolan et al., 2020)
Abscisic acid (ABA)	(Marion-Poll and Leung, 2007; Cutler et al., 2010; Ng et al., 2014; Chen et al., 2020)
Jasmonic acid (JA)	(Chini et al., 2009; Wasternack and Song, 2017; Ruan et al., 2019)
Ethylene	(Bleecker and Kende, 2000; Lin et al., 2009; Ju and Chang, 2015; Dubois et al., 2018)
Salicylic acid (SA)	(Seyfferth and Tsuda, 2014; Janda and Ruelland, 2015; Maruri-López et al., 2019; Ding and Ding, 2020)
Strigolactone (SL)	(Al-Babili and Bouwmeester, 2015; Waters et al., 2017; Jia et al., 2019)

Table S2. Proteins encoded by genes described in this review and in the table S3.

Hormone	Process	Protein		Description	Reference
Auxin	Synthesis	Cytochrome P450s CYP79B2 and CYP79B3		Enzymes that convert tryptophan (Trp) to indole-3-acetaldoxime (IAOx)	(Zhao et al., 2002)
		WEAK ETHYLENE INSENSITIVE2/ANTHRANILATE SYNTHASE alpha1 (WEI2/ASA1)	WEI2/ASA1	ASA1 is the alpha subunit of the anthranilate synthase enzyme, which function in the rate-limiting step Trp biosynthesis	(Stepanova et al., 2005)
		WEI7/ANTHRANILATE SYNTHASE beta1 (ASB1)	WEI7/ASB1	ASB11 is the beta subunit of the anthranilate synthase enzyme, which participates in the rate-limiting step of Trp biosynthesis	
		TRYPTOPHAN AMINOTRANSFERASE OF ARABIDOPSIS1	WEI8/TAA1	Enzymes that synthetize indole-3-pyruvate (IPA), an IAA precursor, from L-Trp	(Tao et al., 2008; Brumos et al., 2014)
		TRYPTOPHAN AMINOTRANSFERASE RELATED 2	TAR1 and TAR2		
		YUCCA	YUC	Enzyme that produces IAA from IPA	(Dharmasiri et al., 2005; Quint and Gray, 2006; Brumos et al., 2014)
	Signaling	TRANSPORT INHIBITOR RESPONSE 1	TIR1	F-box protein that functions as auxin receptor and form part of the SCF ubiquitin ligase complex.	
		AUXIN SIGNALING FBOX PROTEIN	AFB		
		AUXIN BINDING PROTEIN 1	ABP1	Auxin receptor localized at the plasma membrane	(Tromas et al., 2009)
		AUXIN/INDOLE ACETIC ACID	AUX/IAA	TFs that act as repressors of the auxin signaling pathway through the dimerization with *ARFs.*	(Luo et al., 2018)
		AUXIN RESPONSE FACTOR	ARF	TFs that regulate the transcript accumulation of target genes that have auxin response elements (AuxREs) on their regulatory regions.	(Roosjen et al., 2018)
	Transport	PIN-FORMED	PIN	Auxin efflux transporters	(Swarup and Bhosale, 2019)
		ARABIDOPSIS THALIANA ATPBINDING CASSETTE B	ABCB		
		AUXIN TRANSPORTER 1 /LIKE-AUXS	AUX1/ LAXS	Auxin influx transporters	
		PINOID	PID	Serine/threonine kinase that regulate PIN localization	(Raftopoulou, 2004)
		PIN-LIKES	PILS	Putative auxin carriers that control the intracellular auxin accumulation at the endoplasmic reticulum.	(Feraru et al., 2019; Sun et al., 2020)
	Conjugation	GRETCHEN HAGEN 3	GH3	IAA-amido synthetase that catalyzes the conjugation of IAA with amino acids.	(Staswick et al., 2005)

Table S2. Proteins encoded by genes described in this review and in the table S3 (continuation)

Hormone	Process	Protein		Description	Reference
CK	Synthesis	ISOPENTENYL TRANSFERASE	IPT	Enzyme that catalyzes the first step of isoprenoid CK biosynthesis.	(Kamada-Nobusada and Sakakibara, 2009)
	Signaling	ARABIDOPSIS HISTIDINE-KINASE RECEPTORS 2, 3 and 4	AHK2,3,4	Receptors with kinase function that perceive CK, autophosphorylate in response to the CK signal and transfer it to the nucleus via a multi phosphorelay system.	(Zürcher and Müller, 2016)
	Signaling	ARABIDOPSIS HISTIDINE PHOSPHOTRANSFER	AHP	Protein that receives the phosphate of the AHK receptors and donate it to the ARR members	
		TYPE-B ARABIDOPSIS RESPONSE REGULATORS	B-ARR	Positive regulators of CK signaling that transcriptionally regulate the accumulation of CK responsive genes, including A-type ARRs	(Argyros et al., 2008)
		TYPE-A ARABIDOPSIS RESPONSE REGULATORS	A-ARR	Negative-feedback regulators of CK signaling	(Perilli et al., 2010)
	Catabolism	CYTOKININ OXIDASES/ DEHYDROG ENASES	CKX	Catalyzes the irreversible degradation of cytokinins	(Schmülling et al., 2003)
GA	Synthesis	ENT-COPALYL DIPHOSPHATE SYNTHASE	GA1/ CPS	Catalyzes the conversion of geranylgeranyl diphosphate to ent-copalyl diphosphate (a GA precursor)	(Sun Tai ping and Kamiya, 1994)
	Signaling	GIBBERELLIN INSENSITIVE DWARF 1	GID1	GA nuclear receptor	(Nakajima et al., 2006)
		SLEEPY	SLY	F-box protein that is part of the SCF^{SLY1} E3 ligase complex required for the ubiquitination and degradation of DELLA proteins	(Dill et al., 2004)
		GA INSENSITIVE	GAI	DELLAs (Aspartic acid–Glutamic acid–Leucine–Leucine–Alanine) proteins that function as transcriptional negative regulators of GA signaling.	(Vera-Sirera et al., 2016)
		REPRESSOR OF GAI	RGA		
		RGA-LIKE1, 2 and 3	RGL1,2 and 3		
BR	Synthesis	CONSTITUTIVE PHOTOMORPHOGENESIS AND DWARFISM	CPD	Cytochrome P450 monooxygenase that participates in the oxidation of BR intermediates	(Ohnishi et al., 2012)
		DEETIOLATED2	DET2	Steroid 5 alpha-reductases that act in the first step of BR synthesis	(Fujioka et al., 1997)
		DWARF4/ C-22 hydroxylase	DWF4	Catalyzes diverse hydroxylation steps in the BR biosynthesis	(Choe et al., 1998)
	Signaling	BRASSINOSTEROID INSENSITIVE1	BRI1	BR plasma membrane receptor	(Friedrichsen et al., 2000)

Table S2. Proteins encoded by genes described in this review and in the table S3 (continuation)

Hormone	Process	Protein		Description	Reference
BR	Signaling	BRI1-ASSOCIATED RECEPTOR KINASE1/SOMATIC EMBRYOGENESIS RECEPTOR KINASE3	BAK1/SERK3	BAK1 form a complex with BRI1 (BRI1/BAK1) to initiate a signalling cascade that activates BZR1/BES2 and BZR2/BES1	(Sun et al., 2010)
		BRASSINOSTEROID SIGNALING KINASE	BSK	Receptor-like cytoplasmic kinase localized on plasma membrane	(Shi et al., 2013)
		BRASSINOSTEROID INSENSITIVE2	BIN2	Negative regulator of BR signaling, it phosphorylates BZR1/BES2 and BZR2/BES1 preventing their function.	(Yan et al., 2009b)
		BIN2 like1/2	BIL1/2	Homologs of BIN2 that act redundantly during BR signaling	
		BRASSINAZOLE RESISTANT1/ BRI1 EMS suppressor 2	BZR1/BES2	Positive regulators of the BR signalling pathway	(Wang et al., 2002)
		BRASSINAZOLE RESISTANT2/ BRI1 EMS suppressor 1	BZR2/ BES1		
ABA	Synthesis	ZEAXANTHIN EPOXIDASE/ ABSCISIC ACID DEFICIENT1	ZEP/ABA1	ABA1 functions in the first step of the ABA biosynthesis, converts zeaxanthin into violaxanthin via the intermediate antheraxanthin.	(Finkelstein, 2013)
		SHORT-CHAIN DEHYDROGENASE/RE DUCTASE/ ABSCISIC ACID DEFICIENT2	SDR/ABA2	Catalyzes the production of xanthoxin to abscisic aldehyde	
		MOLYBDENUM COFACTOR SULFURASE	MCS/ABA3	Enzyme required in the synthesis of the sulfurated form of the molybdenum cofactor (MoCo), which is necessary for the function of Mo enzymes such as aldehyde oxidase (AO) and xanthine dehydrogenase (XDH).	(Watanabe et al., 2018)
	Signaling	PYRABACTIN RESISTANCE/PYRABACTIN RESISTANCE–LIKE/REGULATORY COMPONENTS OF ABA RECEPTORS	PYR/PYL/RCAR	Family of ABA receptors	(Dittrich et al., 2019)
		ABA-INSENSITIVE1 AND 2	ABI1 and ABI2	Protein phosphatases of type 2C (PP2Cs), which function as coreceptors and negative regulators of ABA signaling	(Merlot et al., 2001)Merlot et al., 2001
		SNF1-RELATED PROTEIN KINASES2S	SnRK2s	Kinases that phosphorylate AREB/ABF-type bZIP TFs to activate ABA-responsive genes expression	(Finkelstein, 2013)
		BRASSINOSTEROID INSENSITIVE1-LIKE RECEPTOR KINASE 1/3	BRL1/3	BR receptors homologs to BRI1	Caño-Delgado et al., 2004

Table S2. Proteins encoded by genes described in this review and in the table S3 (continuation)

Hormone	Process	Protein		Description	Reference
ABA	Signaling	ABA-INSENSITIVE3 AND 5	ABI3, ABI4 and ABI5	ABA response-related TFs and targets of SnRK2s, that functions as positive regulators of the ABA response	(Finkelstein, 2013; Yang et al., 2017)
		HYPERSENSITIVE TO ABA1	HAB1	PP2Cs that acts as negative regulator of ABA signaling	Saez et al., 2004
Ethylene	Synthesis	ETHYLENE OVERPRODUCER	ETO1	Regulator of the ethylene pathway, which functions as a substrate-specific adapter between ACS enzymes to ubiquitin ligase complexes, regulating ACS ubiquitination and degradation.	(Wang et al., 2004)
		1-AMINOCYCLOPROPANE-1- CARBOXYLATE (ACC) SYNTHASE	ETO2/ACS	Enzyme that catalyzes the synthesis of the ethylene precursor ACC from S-Adenosyl methionine (SAM)	(Wang et al., 2002)
	Signaling	ETHYLENE RESPONSE 1/2	ETR1/2	Ethylene receptors that are negative regulators of the signaling transduction pathway	(Chen et al., 2005)
		ETHYLENE INSENSITIVE 4	EIN4		
		ETHYLENE RESPONSE SENSOR 1/2	ERS1/2		
		ETHYLENE INSENSITIVE2	EIN2	Central component of ethylene signaling that acts downstream of ethylene receptors. When the ethylene signaling is derepressed, EIN2 is cleaved and its processed C-terminal product (EIN2C) is translocated to the nucleus to stabilize EIN3 and EIL1	
		CONSTITUTIVE RESPONSE 1	CTR1	Kinase that acts as negative regulator of ethylene signaling via inactivation of EIN2	
		ETHYLENE INSENSITIVE3/EIN3- LIKE	EIN3/EIL 1	TFs that are positive regulators of ethylene signaling and stimulate the transcription of TFs as *E*THYLENE *R*ESPONSE *F*ACTOR (ERF1)	
JA	Synthesis	ALLENE OXIDE SYNTHASE	AOS	Cytochrome P450 enzyme that participates in the first step in the conversion of α-linolenic acid to JA	(Laudert and Weiler, 1998)
	Signaling	CORONATINE INSENSITIVE 1	COI1	F-box protein that acts a JA receptor and form the SCF^{COI1} E3 ubiquitin ligase complex to recruit JAZ proteins for degradation.	(Yan et al., 2009a)
		JASMONATE ZIM DOMAIN PROTEINS	JAZ	Negative regulators of JA signaling impeding the function of transcriptional activators in the absence of JA-Ile	(Thines et al., 2007)
		NOVEL INTERACTOR OF JAZ	NINJA	Transcriptional repressor of JA signaling, it is recruited by JAZ proteins.	(Wasternack and Hause, 2013)
		Helix- loop-helix (bHLH) proteins	MYC2	TF that interacts with the Mediator Complex and recruit the RNA-pol II to JA-responsive genes	(Zhai and Li, 2019)

Table S2. Proteins encoded by genes described in this review and in the table S3 (continuation)

Hormone	Process	Protein		Description	Reference
SA	Signaling	NONEXPRESSOR OF PR GENES 1/3/4	NPR1/3/4	SA receptors	(Zhai and Li, 2019)
SL	Synthesis	CAROTENOID CLEAVAGE DIOXYGENASE 7	CCD 7/ MAX3	Participates in the conversion of 9-cis-β-carotene into 9-cis-β-apo-10'-Carotenal	(Al-Babili and Bouwmeester, 2015)
		CAROTENOID CLEAVAGE DIOXYGENASE 8	CCD 8/ MAX4	Participates in the conversion of 9-cis-β-apo-10'-Carotenal into carlactone	
		CYTOCHROME P450 MONOOXYGENASE	MAX1	Catalyzes the conversion of carlactone in SL parent molecule	
	Signaling	MORE AXILLARY GROWTH 1	MAX2	F-box protein, part of the SCF–type ubiquitin ligase complex	

Table S3. Primary root phenotype of the loss and gain-of-function (LoF and GoF) mutants of genes that participate in hormone homeostasis compared to WT

Hormone	Process	Mutant	Type of mutant	Phenotype	Reference
AUXIN	Synthesis	*cyp79b2 cyp79b3*	LoF	Short	(Zhao et al., 2002)
		CYP79B2ox	OE	Long	
		tir7-1 tir2-1 (asa1 taa1)	LoF	Short	(Yamada et al., 2009)
		wei8 tar2		Short	(He et al., 2011)
		wei8-1 tar1-1 tar2-1	LoF	Without root	(Stepanova et al., 2008)
		yuc3 yuc5 yuc7 yuc8 yuc9	LoF	Short	(Chen et al., 2014)
		YUC1	OE	Short	(Cheng et al., 2006)
		YUC2	OE	Short	
		YUC4	OE	Short	
		YUC6	OE	Short	
	Conjugation	YDK1-D/GH3	OE	Short	(Takase et al., 2004)
	Signaling	*ARF19*	OE	Short	(Okushima et al., 2005)
		arf10 arf16	LoF	Short, agravitopic root	(Wang et al., 2005)
		axr3/iaa17	GoF	Short	(Leyser et al., 1996)
		IAA20	OE	Short	(Sato and Yamamoto, 2008)
		IAA30	OE	Short	
		IAA31	OE	Short	
	Transport	*pin1 pin2*	LoF	Short	(Blilou et al., 2005)
		pin1 pin3	LoF	Short	
		pin2 pin3	LoF	Short	
		pin2 pin4	LoF	Short	
		pin3 pin7	LoF	Short	
		pin3pin4	LoF	Long	
		pin1pin2pin3	LoF	Short	
		pin1pin2pin7	LoF	Short	
		pin2pin4pin7	LoF	Short	
		pin2pin3pin7	LoF	Short	
		pin2pin3pin4	LoF	Short	
		pin3pin4pin7	LoF	Long	
		PIN1	OE	Long	(Ganguly et al., 2010)
		PIN2	OE	Long	
		PIN3	OE	Long	
		PIN7	OE	Long	
		PIN5	OE	Short	(Di Mambro et al., 2019)
		pin5-3	LoF	Long	
		pin6-2	LoF	Long	(Cazzonelli et al., 2013)

Table S3. Primary root phenotype of the loss and gain-of-function (LoF and GoF) mutants of genes that participate in hormone homeostasis compared to WT

Hormone	Process	Mutant	Type of mutant	Phenotype	Reference
AUXIN	Transport	PIN6	OE	Short	(Cazzonelli et al., 2013)
		ABP1	LoF	Short	(Tromas et al., 2009)
		pils6-1	LoF	Long	(Feraru et al., 2019)
		pils6-2	LoF	Long	
		PIL6	OE	Short	
		PIL5	OE	Short	(Sun et al., 2020)
		pils2 pils3 pils5	LoF	Short	
		mrp5 (ABC transporter)	LoF	Short	(Gaedeke et al., 2001)
CK	Synthesis	*ipt3 ipt5 ipt7*	LoF	Long	(Miyawaki et al., 2006; Dello Ioio et al., 2007)
		ipt1 ipt3 ipt5 ipt7	LoF	Long	(Miyawaki et al., 2006)
		IPT8	OE	Short	(Wang et al., 2015)
	Catabolism	CKX1	OE	Long	(Werner et al., 2001, 2003)
		CKX2	OE	Long	(Werner et al., 2003)
		CKX3	OE	Long	
		CKX4	OE	Long	
	Signaling	*arr1-4*	LoF	Long	(Mason et al., 2005; Dello Ioio et al., 2007)
		arr12-1	LoF	Long	(Mason et al., 2005)
		arr1 arr10	LoF	Long	
		arr1 arr12	LoF	Long	
		arr1 arr18	LoF	Long	
		arr11 arr12	LoF	Long	
		arr12 arr18	LoF	Long	
		arr1 arr2 arr11	LoF	Long	
		arr1 arr2 arr12	LoF	Long	
		arr1 arr10 arr11	LoF	Long	
		arr1 arr10 arr12	LoF	Long	
		arr1 arr11 arr12	LoF	Long	
		arr10 arr11 arr12	LoF	Long	
		arr2 arr11 arr12	LoF	Long	
		arr1-3 arr10-5	LoF	Long	(Argyros et al., 2008)
		arr10-5 arr12-1	LoF	Long	
		arr1-3 arr10-2 arr12-1	LoF	Long	
		arr1-4 arr10-5	LoF	Short	(Ishida et al., 2008)
		arr1-4 arr10-5 arr12-1	LoF	Short	
		arr3 arr4	LoF	Short	(To et al., 2004)
		arr4 arr5	LoF	Short	

Table S3. Primary root phenotype of the loss and gain-of-function (LoF and GoF) mutants of genes that participate in hormone homeostasis compared to WT (continuation)

Hormone	Process	Mutant	Type of mutant	Phenotype	Reference
CK	Signaling	*arr3 arr4 arr8 arr9*	LoF	Short	(To et al., 2004)
		arr3 arr4 arr5 arr6 arr8 arr9	LoF	Short	
		ARR4	OE	Reduced PR growth inhibition in response to CK	(To et al., 2007)
		ARR5	OE		
		ARR6	OE		
		ARR7	OE		
		ARR9	OE		
		ahk3-3	LoF	Long	(Dello Ioio et al., 2007)
		cre1-2/ahk4	LoF	Long	(Riefler et al., 2006)
		ahk2-5 ahk3-7	LoF	Long	
		ahk2-1 ahk3-1 ahk4-1	LoF	Short	(Ishida et al., 2008)
GA	Synthesis	*ga1-3*	LoF	Short	(Fu and Harberd, 2003)
		ga3ox1	LoF	Short	(Ubeda-Tomás et al., 2009)
		ga3ox2	LoF	Short	
	Signaling	*gai-t6 ga1-3*	LoF	PR shorter than WT, but longer than *ga1-3* single mutant	(Fu and Harberd, 2003)
		rga-24 ga1-3	LoF		
		gai-t6 rga-24 ga1-3	LoF	Revert the *ga1-3* phenotype	
BR	Synthesis	*det-2*	LoF	Short	(Li et al., 2020)
		cbb3 (cpd)	LoF	Short	(Müssig et al., 2003)
		dwf1-6 (cbb1)	LoF	Short	
		dwf4 (psc1)	LoF	Short	(Ren et al., 2009; Chaiwanon and Wang, 2015)
	Signaling	*BRI1*	OE	Short	(González-García et al., 2011)
		bri1-116	LoF	Short	(Chaiwanon and Wang, 2015)
		bak1-1	LoF	Short	(Li et al., 2002)
		bak1-1D	GoF	Long	
		BES1-RNAi	LoF	Short	(Li et al., 2020)
		bri1 brl1 brl3	LoF	Short	Kang et al., 2017
		bin2-1	GoF	Short	(Li et al., 2020)
		bin2-3bil1bil2	LoF	Long	
		bzr1-1D	GoF	Short	(González-García et al., 2011)
		bri1-116 bzr1-1D	LoF/GoF	Rescue the short PR phenotype of *bri1-116* and *dwf4*	(Chaiwanon and Wang, 2015)

Table S3. Primary root phenotype of the loss and gain-of-function (LoF and GoF) mutants of genes that participate in hormone homeostasis compared to WT (continuation)

Hormone	Process	Mutant	Type of mutant	Phenotype	Reference
ABA	Synthesis	*aba1-6*	LoF	Short	(Ha et al., 2018)
	Signaling	*abi4*	LoF	Long under ABA treatment	(Munguía-Rodríguez et al., 2020)
		HAB1		Long under ABA treatment	(Rodrigues et al., 2009)
		hab1-1 abi1-2	LoF	Short under ABA treatment	
		hab1-1 abi1-2 abi2-2	LoF	Short under ABA treatment	
		hab1-1 abi1-2 pp2ca-1	LoF		
		pyl8	LoF	Long under ABA treatment	(Antoni et al., 2013)
		pyl8-1pyl9	LoF		(Xing et al., 2016)
		PYL9	OE	Short under ABA treatment	
		abi1-2 hab1-1 pp2ca-1	LoF		(Antoni et al., 2013)
		pyl8 hab1-1 abi1-2	LoF		
		pyl8 pp2ca-1 abi1-2	LoF		
Ethylene	Synthesis	*eto1-2*	LoF	Short	(He et al., 2011)
	Signaling	*etr1-3*	LoF	Resistant to ACC root growth inhibition	(Růžička et al., 2007)
		ein2	LoF		
		ctr11-1	LoF	Short	(He et al., 2011)
		EIN3	OE	Short	
JA	Signaling	*coi1-1*	LoF	Insensitive to JA root growth inhibition. Long PR under JA treatment	(Chen et al., 2011; Raya-González et al., 2012; Gasperini et al., 2015)
		jin1-1 (myc2)	LoF	Insensible to JA PR inhibition	(Dombrecht et al., 2007; Chen et al., 2017)
		jin1-9 (myc2)	LoF		
		ninja	LoF	Short	(Acosta et al., 2013; Gasperini et al., 2015)
		ninja aos	LoF	Short	(Gasperini et al., 2015)
		myc2-322B	LoF	Short	
		myc2-322B aos	LoF	Short	
		ninja-1 myc2-322B coi1-1	LoF	Long under JA treatment	
		ninja-1 myc2-322B aos	LoF	Short	

Table S3. Primary root phenotype of the loss and gain-of-function (LoF and GoF) mutants of genes that participate in hormone homeostasis compared to WT (continuation)

Process	Mutant	Type of mutant	Phenotype	Reference
CROSSTALK	*axr2/iaa7*	GoF	Long under ABA treatment	(Li et al., 2017)
	arf2-101	LoF	Short under ABA treatment	(Wang et al., 2011)
	ARF2	OE	Long under ABA treatment	
	YUC4	OE	Short under ABA treatment	(Munguía-Rodríguez et al., 2020)
	abi4 YUC4	LoF/OE	Restaures the sensitibity to ABA inhibition similarly to WT	
	pin1-1	LoF	Reduced sensitivity to the stimulatory effect of PR growth by shoot-ABA treatment. Less sensitive to BR treatment.	(Li et al., 2005; Xie et al., 2020)
	tir1-1	LoF	Long under ABA or ACC treatment	(Thole et al., 2014)
	aux1-7	LoF	Long under ABA or ACC treatment	
	pin2	LoF	Long under ABA treatment	
	etr1-1	LoF	Long under ABA treatment	
	ein2-1	LoF	Long under ABA or under CK exogenous application	(Thole et al., 2014; Street et al., 2016)
	ein3-1	LoF	Long under ABA treatment	(Thole et al., 2014)
	ein2 aux1	LoF		
	eto1 aux1	LoF		
	ein2-44	LoF		(Ghassemian et al., 2000)
	etr1-4	LoF		
	etr1-2	LoF		
	etr1-3	LoF		
	abi1-1	LoF	Short under ACC exogenous treatment	(Thole et al., 2014)
	abi2-1			
	abi3-1	LoF		
	dwf4	LoF	Short under auxin treatment	(Chaiwanon and Wang, 2015)
	bri1-116	LoF		
	bin2-1	LoF		
	axr2-1/iaa7	GoF	Short under BR exogenous application	(Nakamura et al., 2006)
	axr3-3/iaa17	GoF		
	PILS5	OE	Less sensitive to exogenous 24-Epibrassinolide application	(Sun et al., 2020)
	pils2 pils3 pils5	LoF	Hypersensitive to BL	
	IPT8	OE	Exogenous application of auxin partially reverts the growth inhibition	(Wang et al., 2015)

Table S3. Primary root phenotype of the loss and gain-of-function (LoF and GoF) mutants of genes that participate in hormone homeostasis compared to WT (continuation)

Process	Mutant	Type of mutant	Phenotype	Reference
CROSSTALK	yuc1	LoF	Long PR under CK treatment	(Di et al., 2016)
	yuc2	LoF		
	yuc4	LoF		
	yuc6	LoF		
	yuc7	LoF		
	yuc10	LoF		
	yuc11	LoF		
	aux1	LoF	Long PR under CK application, insensitive to exogenous ACC application	(Street et al., 2016)
	pin5-3	LoF	Insensitive to CK PR inhibition	(Di Mambro et al., 2019)
	pin2/eir1	LoF	Insensitive to ACC inhibition	(Růžička et al., 2007)
	PIN1	OE		
	PIN6	OE		(Cazzonelli et al., 2013)
	wei2-1	LoF	Long PR under ACC exogenous	(Stepanova et al., 2005, 2008)
	wei7-4	LoF		
	wei8-1	LoF		
	wei8-2	LoF		
	arf19-101	LoF	More resistant to PR growth inhibition of ACC exogenous application	(Li et al., 2006)
	arf7-201	LoF		
	arf19-101 arf7-201	LoF		
	jin1-9 (myc2)	LoF	Less sensitive to the synthetic auxin transport inhibitor TIBA	(Dombrecht et al., 2007)
	coi1-2 psc1 (dwf4)	LoF	Reverts the insensitivity in PR growth of *coi1* to JA application and the PR inhibition by JA is suppressed with the application of epibrassinolide	(Ren et al., 2009)
	rga-24	LoF	Increase the PR growth under ACC exogenous treatment	(Achard et al., 2003)
	gait-6 rga-24	LoF		
	ga1-3		Short under JA exogenous treatment	(Hou et al., 2010)
	ga1 hy1-101	LoF	Short	
	ga1 rga28	LoF	Highly sensitive to JA inhibition	
	max1	LoF	Less sensitivity to the exogenous application of MeJA	(Dombrecht et al., 2007)

REFERENCES OF SUPPLEMENTARY MATERIAL

Achard, P., Vriezen, W. H., Van Der Straeten, D., and Harberd, N. P. (2003). Ethylene Regulates Arabidopsis Development via the Modulation of DELLA Protein Growth Repressor Function. *Plant Cell* 15, 2816–2825. doi:10.1105/tpc.015685.

Acosta, I. F., Gasperini, D., Chetelat, A., Stolz, S., Santuari, L., and Farmer, E. E. (2013). Role of NINJA in root jasmonate signaling. *Proc. Natl. Acad. Sci.* 110, 15473–15478. doi:10.1073/pnas.1307910110.

Al-Babili, S., and Bouwmeester, H. J. (2015). Strigolactones, a Novel Carotenoid-Derived Plant Hormone. *Annu. Rev. Plant Biol.* 66, 161–186. doi:10.1146/annurev-arplant-043014-114759.

Antoni, R., Gonzalez-Guzman, M., Rodriguez, L., Peirats-Llobet, M., Pizzio, G. A., Fernandez, M. A., et al. (2013). PYRABACTIN RESISTANCE1-LIKE8 plays an important role for the regulation of abscisic acid signaling in root. *Plant Physiol.* 161, 931–941. doi:10.1104/pp.112.208678.

Argyros, R. D., Mathews, D. E., Chiang, Y. H., Palmer, C. M., Thibault, D. M., Etheridge, N., et al. (2008). Type B response regulators of Arabidopsis play key roles in cytokinin signaling and plant development. *Plant Cell* 20, 2102–2116. doi:10.1105/tpc.108.059584.

Bleecker, A. B., and Kende, H. (2000). Ethylene: A gaseous signal molecule in plant. *Annu. Rev. Cell Dev. Biol.* 16, 1–18. doi:10.1146/annurev.cellbio.16.1.1.

Blilou, I., Xu, J., Wildwater, M., Willemsen, V., Paponov, I., Frimi, J., et al. (2005). The PIN auxin efflux facilitator network controls growth and patterning in Arabidopsis roots. *Nature* 433, 39–44. doi:10.1038/nature03184.

Brumos, J., Alonso, J. M., and Stepanova, A. N. (2014). Genetic aspects of auxin biosynthesis and its regulation. *Physiol. Plant.* 151, 3–12. doi:10.1111/ppl.12098.

Cazzonelli, C. I., Vanstraelen, M., Simon, S., Yin, K., Carron-Arthur, A., Nisar, N., et al. (2013). Role of the Arabidopsis PIN6 Auxin Transporter in Auxin Homeostasis and Auxin-Mediated Development. *PLoS ONE* 8, e70069. doi:10.1371/journal.pone.0070069.

Chaiwanon, J., and Wang, Z.-Y. (2015). Spatiotemporal Brassinosteroid Signaling and Antagonism with Auxin Pattern Stem Cell Dynamics in Arabidopsis Roots HHS Public Access Author manuscript. *Curr Biol* 25, 1031–1042. doi:10.1016/j.cub.2015.02.046.

Chen, K., Li, G., Bressan, R. A., Song, C., Zhu, J., and Zhao, Y. (2020). Abscisic acid dynamics, signaling, and functions in plants. *J. Integr. Plant Biol.* 62, 25–54. doi:10.1111/jipb.12899.

Chen, Q., Dai, X., De-Paoli, H., Cheng, Y., Takebayashi, Y., Kasahara, H., et al. (2014). Auxin overproduction in shoots cannot rescue auxin deficiencies in arabidopsis roots. *Plant Cell Physiol.* 55, 1072–1079. doi:10.1093/pcp/pcu039.

Chen, Q., Sun, J., Zhai, Q., Zhou, W., Qi, L., Xu, L., et al. (2011). The basic helix-loop-helix transcription factor myc2 directly represses plethora expression during jasmonate-mediated modulation of the root stem cell niche in arabidopsis. *Plant Cell* 23, 3335–3352. doi:10.1105/tpc.111.089870.

Chen, Y. F., Etheridge, N., and Schaller, G. E. (2005). *Ethylene signal transduction*. Oxford University Press doi:10.1093/aob/mci100.

Chen, Y., Wang, Y., Huang, J., Zheng, C., Cai, C., Wang, Q., et al. (2017). Salt and methyl jasmonate aggravate growth inhibition and senescence in Arabidopsis seedlings via the JA signaling pathway. *Plant Sci.* 261, 1–9. doi:10.1016/j.plantsci.2017.05.005.

Cheng, Y., Dai, X., and Zhao, Y. (2006). Auxin biosynthesis by the YUCCA flavin monooxygenases controls the formation of floral organs and vascular tissues in Arabidopsis. *Genes Dev.* 20, 1790–1799. doi:10.1101/gad.1415106.

Chini, A., Boter, M., and Solano, R. (2009). Plant oxylipins: COI1/JAZs/MYC2 as the core jasmonic acid-signalling module. *FEBS J.* 276, 4682–4692. doi:10.1111/j.1742-4658.2009.07194.x.

Choe, S., Dilkes, B. P., Fujioka, S., Takatsuto, S., Sakurai, A., and Feldmann, K. A. (1998). The *DWF4* Gene of Arabidopsis Encodes a Cytochrome P450 That Mediates Multiple 22α-Hydroxylation Steps in Brassinosteroid Biosynthesis. *Plant Cell* 10, 231–243. doi:10.1105/tpc.10.2.231.

Chung, Y., and Choe, S. (2013). The Regulation of Brassinosteroid Biosynthesis in Arabidopsis. *Crit. Rev. Plant Sci.* 32, 396–410. doi:10.1080/07352689.2013.797856.

Cutler, S. R., Rodriguez, P. L., Finkelstein, R. R., and Abrams, S. R. (2010). Abscisic Acid: Emergence of a Core Signaling Network. *Annu. Rev. Plant Biol.* 61, 651–679. doi:10.1146/annurev-arplant-042809-112122.

Davière, J. M., and Achard, P. (2013). Gibberellin signaling in plants. *Dev. Camb.* 140, 1147–1151. doi:10.1242/dev.087650.

Dello Ioio, R., Linhares, F. S., Scacchi, E., Casamitjana-Martinez, E., Heidstra, R., Costantino, P., et al. (2007). Cytokinins Determine Arabidopsis Root-Meristem Size by Controlling Cell Differentiation. *Curr. Biol.* 17, 678–682. doi:10.1016/j.cub.2007.02.047.

Dharmasiri, N., Dharmasiri, S., and Estelle, M. (2005). The F-box protein TIR1 is an auxin receptor. *Nature* 435, 441–445. doi:10.1038/nature03543.

Di, D.-W., Wu, L., Zhang, L., An, C.-W., Zhang, T.-Z., Luo, P., et al. (2016). Functional roles of Arabidopsis CKRC2/YUCCA8 gene and the involvement of PIF4 in the regulation of auxin biosynthesis by cytokinin. *Sci. Rep.* 6, 36866. doi:10.1038/srep36866.

Di Mambro, R., Svolacchia, N., Dello Ioio, R., Pierdonati, E., Salvi, E., Pedrazzini, E., et al. (2019). The Lateral Root Cap Acts as an Auxin Sink that Controls Meristem Size. *Curr. Biol.* 29, 1199-1205.e4. doi:10.1016/j.cub.2019.02.022.

Dill, A., Thomas, S. G., Hu, J., Steber, C. M., and Sun, T. P. (2004). The arabidopsis F-box protein SLEEPY1 targets gibberellin signaling repressors for gibberellin-induced degradation. *Plant Cell* 16, 1392–1405. doi:10.1105/tpc.020958.

Ding, P., and Ding, Y. (2020). Stories of Salicylic Acid: A Plant Defense Hormone. *Trends Plant Sci.* 25, 549–565. doi:10.1016/j.tplants.2020.01.004.

Dittrich, M., Mueller, H. M., Bauer, H., Peirats-Llobet, M., Rodriguez, P. L., Geilfus, C. M., et al. (2019). The role of Arabidopsis ABA receptors from the PYR/PYL/RCAR family in stomatal acclimation and closure signal integration. *Nat. Plants* 5, 1002–1011. doi:10.1038/s41477-019-0490-0.

Dombrecht, B., Gang, P. X., Sprague, S. J., Kirkegaard, J. A., Ross, J. J., Reid, J. B., et al. (2007). MYC2 differentially modulates diverse jasmonate-dependent functions in Arabidopsis. *Plant Cell* 19, 2225–2245. doi:10.1105/tpc.106.048017.

Dubois, M., Van den Broeck, L., and Inzé, D. (2018). The Pivotal Role of Ethylene in Plant Growth. *Trends Plant Sci.* 23, 311–323. doi:10.1016/j.tplants.2018.01.003.

Feraru, E., Feraru, M. I., Barbez, E., Waidmann, S., Sun, L., Gaidora, A., et al. (2019). PILS6 is a temperature-sensitive regulator of nuclear auxin input and organ growth in Arabidopsis thaliana. *Proc. Natl. Acad. Sci. U. S. A.* 116, 3893–3898. doi:10.1073/pnas.1814015116.

Finkelstein, R. (2013). Abscisic Acid Synthesis and Response. *Arab. Book* 11, e0166. doi:10.1199/tab.0166.

Friedrichsen, D. M., Joazeiro, C. A. P., Li, J., Hunter, T., and Chory, J. (2000). Brassinosteroid-insensitive-1 is a ubiquitously expressed leucine-rich repeat receptor serine/threonine kinase. *Plant Physiol.* 123, 1247–1255. doi:10.1104/pp.123.4.1247.

Fu, X., and Harberd, N. P. (2003). Auxin promotes Arabidopsis root growth by modulating gibberellin response. *Nature* 421, 740–743. doi:10.1038/nature01387.

Fu, Z. Q., and Dong, X. (2013). Systemic Acquired Resistance: Turning Local Infection into Global Defense. *Annu. Rev. Plant Biol.* 64, 839–863. doi:10.1146/annurev-arplant-042811-105606.

Fujioka, S., Li, J., Choi, Y. H., Seto, H., Takatsuto, S., Noguchi, T., et al. (1997). The Arabidopsis deetiolated2 mutant is blocked early in brassinosteroid biosynthesis. *Plant Cell* 9, 1951–1962. doi:10.1105/tpc.9.11.1951.

Gaedeke, N., Klein, M., Kolukisaoglu, U., Forestier, C., Müller, A., Ansorge, M., et al. (2001). The Arabidopsis thaliana ABC transporter AtMRP5 controls root development and stomata movement. *EMBO J.* 20, 1875–1887. doi:10.1093/emboj/20.8.1875.

Ganguly, A., Lee, S. H., Cho, M., Lee, O. R., Yoo, H., and Cho, H. T. (2010). Differential auxin-transporting activities of PIN-FORMED proteins in arabidopsis root hair cells. *Plant Physiol.* 153, 1046–1061. doi:10.1104/pp.110.156505.

Gasperini, D., Chételat, A., Acosta, I. F., Goossens, J., Pauwels, L., Goossens, A., et al. (2015). Multilayered Organization of Jasmonate Signalling in the Regulation of Root Growth. *PLOS Genet.* 11, e1005300. doi:10.1371/journal.pgen.1005300.

Ghassemian, M., Nambara, E., Cutler, S., Kawaide, H., Kamiya, Y., and McCourt, P. (2000). Regulation of abscisic acid signaling by the ethylene response pathway in arabidopsis. *Plant Cell* 12, 1117–1126. doi:10.1105/tpc.12.7.1117.

González-García, M. P., Vilarrasa-Blasi, J., Zhiponova, M., Divol, F., Mora-García, S., Russinova, E., et al. (2011). Brassinosteroids control meristem size by promoting cell cycle progression in Arabidopsis roots. *Development* 138, 849–859. doi:10.1242/dev.057331.

Gupta, R., and Chakrabarty, S. K. (2013). Gibberellic acid in plant: Still a mystery unresolved. *Plant Signal. Behav.* 8. doi:10.4161/psb.25504.

Ha, J.-H., Kim, J.-H., Kim, S.-G., Sim, H.-J., Lee, G., Halitschke, R., et al. (2018). Shoot phytochrome B modulates reactive oxygen species homeostasis in roots via abscisic acid signaling in \textlessi\textgreaterArabidopsis\textless/i\textgreater. *Plant J.* 94, 790–798. doi:10.1111/tpj.13902.

He, W., Brumos, J., Li, H., Ji, Y., Ke, M., Gong, X., et al. (2011). A small-molecule screen identifies L-Kynurenine as a competitive inhibitor of TAA1/TAR activity in Ethylene-Directed Auxin Biosynthesis and root growth in Arabidopsis. *Plant Cell* 23, 3944–3960. doi:10.1105/tpc.111.089029.

Hou, X., Lee, L. Y. C., Xia, K., Yan, Y., and Yu, H. (2010). DELLAs Modulate Jasmonate Signaling via Competitive Binding to JAZs. *Dev. Cell* 19, 884–894. doi:10.1016/j.devcel.2010.10.024.

Hwang, I., and Sakakibara, H. (2006). Cytokinin biosynthesis and perception. *Physiol. Plant.* 126, 528–538. doi:10.1111/j.1399-3054.2006.00665.x.

Ishida, K., Yamashino, T., Yokoyama, A., and Mizuno, T. (2008). Three Type-B Response Regulators, ARR1, ARR10 and ARR12, Play Essential but Redundant Roles in Cytokinin Signal Transduction Throughout the Life Cycle of Arabidopsis thaliana. *Plant Cell Physiol.* 49, 47–57. doi:10.1093/pcp/pcm165.

Janda, M., and Ruelland, E. (2015). Magical mystery tour: Salicylic acid signalling. *Environ. Exp. Bot.* 114, 117–128. doi:10.1016/j.envexpbot.2014.07.003.

Jia, K.-P., Li, C., Bouwmeester, H. J., and Al-Babili, S. (2019). "Strigolactone Biosynthesis and Signal Transduction," in *Strigolactones - Biology and Applications* (Springer International Publishing), 1–45. doi:10.1007/978-3-030-12153-2_1.

Ju, C., and Chang, C. (2015). Mechanistic insights in ethylene perception and signal transduction. *Plant Physiol.* 169, 85–95. doi:10.1104/pp.15.00845.

Kamada-Nobusada, T., and Sakakibara, H. (2009). *Molecular basis for cytokinin biosynthesis*. Phytochemistry doi:10.1016/j.phytochem.2009.02.007.

Kieber, J. J., and Schaller, G. E. (2014). Cytokinins. *Arab. Book* 12, e0168. doi:10.1199/tab.0168.

Kieber, J. J., and Schaller, G. E. (2018). Cytokinin signaling in plant development. *Dev. Camb. Engl.* 145. doi:10.1242/dev.149344.

Laudert, D., and Weiler, E. W. (1998). Allene oxide synthase: A major control point in Arabidopsis thaliana octadecanoid signalling. *Plant J.* 15, 675–684. doi:10.1046/j.1365-313X.1998.00245.x.

Leyser, H. M. O., Pickett, F. B., Dharmasiri, S., and Estelle, M. (1996). Mutations in the AXR3 gene of Arabidopsis result in altered auxin response including ectopic expression from the SAUR-AC1 promoter. *Plant J.* 10, 403–413. doi:10.1046/j.1365-313x.1996.10030403.x.

Leyser, O. (2018). Auxin signaling. *Plant Physiol.* 176, 465–479. doi:10.1104/pp.17.00765.

Li, J., Dai, X., and Zhao, Y. (2006). A role for auxin response factor 19 in auxin and ethylene signaling in Arabidopsis. *Plant Physiol.* 140, 899–908. doi:10.1104/pp.105.070987.

Li, J., Wen, J., Lease, K. A., Doke, J. T., Tax, F. E., and Walker, J. C. (2002). BAK1, an Arabidopsis LRR Receptor-like Protein Kinase, Interacts with BRI1 and Modulates Brassinosteroid Signaling. *Cell* 110, 213–222. doi:10.1016/S0092-8674(02)00812-7.

Li, L., Xu, J., Xu, Z. H., and Xue, H. W. (2005). Brassinosteroids stimulate plant tropisms through modulation of polar auxin transport in Brassica and Arabidopsis. *Plant Cell* 17, 2738–2753. doi:10.1105/tpc.105.034397.

Li, T., Lei, W., He, R., Tang, X., Han, J., Zou, L., et al. (2020). Brassinosteroids regulate root meristem development by mediating BIN2-UPB1 module in Arabidopsis. *PLoS Genet.* 16. doi:10.1371/journal.pgen.1008883.

Li, X., Chen, L., Forde, B. G., and Davies, W. J. (2017). Abscisic acid regulates root elongation through the activities of auxin and ethylene in Arabidopsis thaliana. *Front. Plant Sci.* 8. doi:10.3389/fpls.2017.01493.

Lin, Z., Zhong, S., and Grierson, D. (2009). Recent advances in ethylene research. *J. Exp. Bot.* 60, 3311–3336. doi:10.1093/jxb/erp204.

Ljung, K. (2013). Auxin metabolism and homeostasis during plant development. *Dev. Camb.* 140, 943–950. doi:10.1242/dev.086363.

Luo, J., Zhou, J.-J., and Zhang, J.-Z. (2018). Aux/IAA Gene Family in Plants: Molecular Structure, Regulation, and Function. *Int. J. Mol. Sci.* 19, 259. doi:10.3390/ijms19010259.

Marion-Poll, A., and Leung, J. (2007). "Abscisic Acid Synthesis, Metabolism and Signal Transduction," in *Annual Plant Reviews Volume 24: Plant Hormone Signaling* (Oxford, UK: Blackwell Publishing Ltd), 1–35. doi:10.1002/9780470988800.ch1.

Maruri-López, I., Aviles-Baltazar, N. Y., Buchala, A., and Serrano, M. (2019). Intra and extracellular journey of the phytohormone salicylic acid. *Front. Plant Sci.* 10, 423. doi:10.3389/fpls.2019.00423.

Marzec, M. (2016). Perception and Signaling of Strigolactones. *Front. Plant Sci.* 7. doi:10.3389/fpls.2016.01260.

Mason, M. G., Mathews, D. E., Argyros, D. A., Maxwell, B. B., Kieber, J. J., Alonso, J. M., et al. (2005). Multiple type-B response regulators mediate cytokinin signal transduction in Arabidopsis. *Plant Cell* 17, 3007–3018. doi:10.1105/tpc.105.035451.

Merlot, S., Gosti, F., Guerrier, D., Vavasseur, A., and Giraudat, J. (2001). The ABI1 and ABI2 protein phosphatases 2C act in a negative feedback regulatory loop of the abscisic acid signalling pathway. *Plant J.* 25, 295–303. doi:10.1046/j.1365-313X.2001.00965.x.

Miyawaki, K., Tarkowski, P., Matsumoto-Kitano, M., Kato, T., Sato, S., Tarkowska, D., et al. (2006). Roles of Arabidopsis ATP/ADP isopentenyltransferases and tRNA isopentenyltransferases in cytokinin biosynthesis. *Proc. Natl. Acad. Sci. U. S. A.* 103, 16598–16603. doi:10.1073/pnas.0603522103.

Munguía-Rodríguez, A. G., López-Bucio, J. S., Ruiz-Herrera, L. F., Ortiz-Castro, R., Guevara-García, Á. A., Marsch-Martínez, N., et al. (2020). YUCCA4 overexpression modulates auxin biosynthesis and transport and influences plant growth and development via crosstalk with abscisic acid in Arabidopsis thaliana. *Genet. Mol. Biol.* 43. doi:10.1590/1678-4685-GMB-2019-0221.

Müssig, C., Shin, G. H., and Altmann, T. (2003). Brassinosteroids Promote Root Growth in Arabidopsis. *Plant Physiol.* 133, 1261–1271. doi:10.1104/pp.103.028662.

Nakajima, M., Shimada, A., Takashi, Y., Kim, Y. C., Park, S. H., Ueguchi-Tanaka, M., et al. (2006). Identification and characterization of Arabidopsis gibberellin receptors. *Plant J.* 46, 880–889. doi:10.1111/j.1365-313X.2006.02748.x.

Nakamura, A., Nakajima, N., Goda, H., Shimada, Y., Hayashi, K., Nozaki, H., et al. (2006). Arabidopsis \textlessi\textgreaterAux/IAA\textless/i\textgreater genes are involved in brassinosteroid-mediated growth responses in a manner dependent on organ type. *Plant J.* 45, 193–205. doi:10.1111/j.1365-313X.2005.02582.x.

Ng, L. M., Melcher, K., Teh, B. T., and Xu, H. E. (2014). Abscisic acid perception and signaling: Structural mechanisms and applications. *Acta Pharmacol. Sin.* 35, 567–584. doi:10.1038/aps.2014.5.

Nolan, T. M., Vukasinović, N., Liu, D., Russinova, E., and Yin, Y. (2020). Brassinosteroids: Multidimensional regulators of plant growth, development, and stress responses. in *Plant Cell* (American Society of Plant Biologists), 298–318. doi:10.1105/tpc.19.00335.

Ohnishi, T., Godza, B., Watanabe, B., Fujioka, S., Hategan, L., Ide, K., et al. (2012). CYP90A1/CPD, a Brassinosteroid Biosynthetic Cytochrome P450 of Arabidopsis, Catalyzes C-3 Oxidation*. *J. Biol. Chem.* 287, 31551–31560. doi:10.1074/jbc.M112.392720.

Okushima, Y., Mitina, I., Quach, H. L., and Theologis, A. (2005). AUXIN RESPONSE FACTOR 2 (ARF2): A pleiotropic developmental regulator. *Plant J.* 43, 29–46. doi:10.1111/j.1365-313X.2005.02426.x.

Perilli, S., Moubayidin, L., and Sabatini, S. (2010). The molecular basis of cytokinin function. *Curr. Opin. Plant Biol.* 13, 21–26. doi:10.1016/j.pbi.2009.09.018.

Planas-Riverola, A., Gupta, A., Betegón-Putze, I., Bosch, N., Ibañes, M., and Caño-Delgado, A. I. (2019). Brassinosteroid signaling in plant development and adaptation to stress. *Development* 146, dev151894. doi:10.1242/dev.151894.

Quint, M., and Gray, W. M. (2006). *Auxin signaling*. NIH Public Access doi:10.1016/j.pbi.2006.07.006.

Raftopoulou, M. (2004). PINOID pinpoints auxin. *Nat. Cell Biol.* 6, 1149–1149. doi:10.1038/ncb1204-1149.

Raya-González, J., Pelagio-Flores, R., and López-Bucio, J. (2012). The jasmonate receptor COI1 plays a role in jasmonate-induced lateral root formation and lateral root positioning in Arabidopsis thaliana. *J. Plant Physiol.* 169, 1348–1358. doi:10.1016/j.jplph.2012.05.002.

Ren, C., Han, C., Peng, W., Huang, Y., Peng, Z., Xiong, X., et al. (2009). A leaky mutation in DWARF4 reveals an antagonistic role of brassinosteroid in the inhibition of root growth by jasmonate in Arabidopsis. *Plant Physiol.* 151, 1412–1420. doi:10.1104/pp.109.140202.

Riefler, M., Novak, O., Strnad, M., and Schmülling, T. (2006). Arabidopsis cytokinin receptors mutants reveal functions in shoot growth, leaf senescence, seed size, germination, root development, and cytokinin metabolism. *Plant Cell* 18, 40–54. doi:10.1105/tpc.105.037796.

Rizza, A., and Jones, A. M. (2019). The makings of a gradient: spatiotemporal distribution of gibberellins in plant development. *Curr. Opin. Plant Biol.* 47, 9–15. doi:10.1016/j.pbi.2018.08.001.

Rodrigues, A., Santiago, J., Rubio, S., Saez, A., Osmont, K. S., Gadea, J., et al. (2009). The short-rooted phenotype of the brevis radix mutant partly reflects root abscisic acid hypersensitivity1[C][W][OA]. *Plant Physiol.* 149, 1917–1928. doi:10.1104/pp.108.133819.

Roosjen, M., Paque, S., and Weijers, D. (2018). Auxin Response Factors: output control in auxin biology. *J. Exp. Bot.* 69, 179–188. doi:10.1093/jxb/erx237.

Ruan, J., Zhou, Y., Zhou, M., Yan, J., Khurshid, M., Weng, W., et al. (2019). Jasmonic acid signaling pathway in plants. *Int. J. Mol. Sci.* 20. doi:10.3390/ijms20102479.

Růžička, K., Ljung, K., Vanneste, S., Podhorská, R., Beeckman, T., Friml, J., et al. (2007). Ethylene regulates root growth through effects on auxin biosynthesis and transport-dependent auxin distribution. *Plant Cell* 19, 2197–2212. doi:10.1105/tpc.107.052126.

Santner, A., Calderon-Villalobos, L. I. A., and Estelle, M. (2009). Plant hormones are versatile chemical regulators of plant growth. *Nat. Chem. Biol.* 5, 301–307. doi:10.1038/nchembio.165.

Sato, A., and Yamamoto, K. T. (2008). Overexpression of the non-canonical Aux/IAA genes causes auxin-related aberrant phenotypes in Arabidopsis. *Physiol. Plant.* 133, 397–405. doi:10.1111/j.1399-3054.2008.01055.x.

Schmülling, T., Werner, T., Riefler, M., Krupková, E., and Bartrina Y Manns, I. (2003). Structure and function of cytokinin oxidase/dehydrogenase genes of maize, rice, Arabidopsis and other species. in *Journal of Plant Research* (J Plant Res), 241–252. doi:10.1007/s10265-003-0096-4.

Schwechheimer, C. (2012). Gibberellin signaling in plants - The extended version. *Front. Plant Sci.* 2, 107. doi:10.3389/fpls.2011.00107.

Seyfferth, C., and Tsuda, K. (2014). Salicylic acid signal transduction: The initiation of biosynthesis, perception and transcriptional reprogramming. *Front. Plant Sci.* 5, 697. doi:10.3389/fpls.2014.00697.

Shi, H., Yan, H., Li, J., and Tang, D. (2013). BSK1, a receptor-like cytoplasmic kinase, involved in both BR signaling and innate immunity in *Arabidopsis*. *Plant Signal. Behav.* 8, e24996. doi:10.4161/psb.24996.

Staswick, P. E., Serban, B., Rowe, M., Tiryaki, I., Maldonado, M. T., Maldonado, M. C., et al. (2005). Characterization of an arabidopsis enzyme family that conjugates amino acids to indole-3-acetic acid. *Plant Cell* 17, 616–627. doi:10.1105/tpc.104.026690.

Stepanova, A. N., Hoyt, J. M., Hamilton, A. A., and Alonso, J. M. (2005). A link between ethylene and auxin uncovered by the characterization of two root-specific ethylene-insensitive mutants in arabidopsis. *Plant Cell* 17, 2230–2242. doi:10.1105/tpc.105.033365.

Stepanova, A. N., Robertson-Hoyt, J., Yun, J., Benavente, L. M., Xie, D. Y., Doležal, K., et al. (2008). TAA1-Mediated Auxin Biosynthesis Is Essential for Hormone Crosstalk and Plant Development. *Cell* 133, 177–191. doi:10.1016/j.cell.2008.01.047.

Street, I. H., Mathews, D. E., Yamburkenko, M. V., Sorooshzadeh, A., John, R. T., Swarup, R., et al. (2016). Cytokinin acts through the auxin influx carrier AUX1 to regulate cell elongation in the root. *Development* 143, 3982–3993. doi:10.1242/dev.132035.

Sun, L., Feraru, E., Feraru, M. I., Waidmann, S., Wang, W., Passaia, G., et al. (2020). PIN-LIKES Coordinate Brassinosteroid Signaling with Nuclear Auxin Input in Arabidopsis thaliana. *Curr. Biol.* 30, 1579-1588.e6. doi:10.1016/j.cub.2020.02.002.

Sun Tai ping, and Kamiya, Y. (1994). The Arabidopsis GA1 locus encodes the cyclase ent-kaurene synthetase A of gibberellin biosynthesis. *Plant Cell* 6, 1509–1518. doi:10.1105/tpc.6.10.1509.

Sun, Y., Fan, X. Y., Cao, D. M., Tang, W., He, K., Zhu, J. Y., et al. (2010). Integration of Brassinosteroid Signal Transduction with the Transcription Network for Plant Growth Regulation in Arabidopsis. *Dev. Cell* 19, 765–777. doi:10.1016/j.devcel.2010.10.010.

Swarup, R., and Bhosale, R. (2019). Developmental Roles of AUX1/LAX Auxin Influx Carriers in Plants. *Front. Plant Sci.* 10, 1306. doi:10.3389/fpls.2019.01306.

Takase, T., Nakazawa, M., Ishikawa, A., Kawashima, M., Ichikawa, T., Takahashi, N., et al. (2004). ydk1-D, an auxin-responsive GH3 mutant that is involved in hypocotyl and root elongation. *Plant J.* 37, 471–483. doi:10.1046/j.1365-313X.2003.01973.x.

Tal, I., Zhang, Y., Jørgensen, M. E., Pisanty, O., Barbosa, I. C. R., Zourelidou, M., et al. (2016). The Arabidopsis NPF3 protein is a GA transporter. *Nat. Commun.* 7, 11486. doi:10.1038/ncomms11486.

Tao, Y., Ferrer, J. L., Ljung, K., Pojer, F., Hong, F., Long, J. A., et al. (2008). Rapid Synthesis of Auxin via a New Tryptophan-Dependent Pathway Is Required for Shade Avoidance in Plants. *Cell* 133, 164–176. doi:10.1016/j.cell.2008.01.049.

Teale, W. D., Paponov, I. A., and Palme, K. (2006). Auxin in action: Signalling, transport and the control of plant growth and development. *Nat. Rev. Mol. Cell Biol.* 7, 847–859. doi:10.1038/nrm2020.

Thines, B., Katsir, L., Melotto, M., Niu, Y., Mandaokar, A., Liu, G., et al. (2007). JAZ repressor proteins are targets of the SCFCOI1 complex during jasmonate signalling. *Nature* 448, 661–665. doi:10.1038/nature05960.

Thole, J. M., Beisner, E. R., Liu, J., Venkova, S. V., and Strader, L. C. (2014). Abscisic acid regulates root elongation through the activities of auxin and ethylene in Arabidopsis thaliana. *G3 Genes Genomes Genet.* 4, 1259–1274. doi:10.1534/g3.114.011080.

To, J. P. C., Deruère, J., Maxwell, B. B., Morris, V. F., Hutchison, C. E., Ferreira, F. J., et al. (2007). Cytokinin regulates type-A Arabidopsis response regulator activity and protein stability via two-component phosphorelay. *Plant Cell* 19, 3901–3914. doi:10.1105/tpc.107.052662.

To, J. P. C., Haberer, G., Ferreira, F. J., Deruère, J., Mason, M. G., Schaller, G. E., et al. (2004). Type-A Arabidopsis response regulators are partially redundant negative regulators of cytokinin signaling. *Plant Cell* 16, 658–671. doi:10.1105/tpc.018978.

Tromas, A., Braun, N., Muller, P., Khodus, T., Paponov, I. A., Palme, K., et al. (2009). The AUXIN BINDING PROTEIN 1 is required for differential Auxin responses mediating root growth. *PLoS ONE* 4. doi:10.1371/journal.pone.0006648.

Ubeda-Tomás, S., Federici, F., Casimiro, I., Beemster, G. T. S., Bhalerao, R., Swarup, R., et al. (2009). Gibberellin Signaling in the Endodermis Controls Arabidopsis Root Meristem Size. *Curr. Biol.* 19, 1194–1199. doi:10.1016/j.cub.2009.06.023.

Vera-Sirera, F., Gomez, M. D., and Perez-Amador, M. A. (2016). “DELLA Proteins, a Group of GRAS Transcription Regulators that Mediate Gibberellin Signaling,” in *Plant Transcription Factors: Evolutionary, Structural and Functional Aspects* (Elsevier Inc.), 313–328. doi:10.1016/B978-0-12-800854-6.00020-8.

Vukašinović, N., and Russinova, E. (2018). BRexit: Possible Brassinosteroid Export and Transport Routes. *Trends Plant Sci.* 23, 285–292. doi:10.1016/j.tplants.2018.01.005.

Wang, J. W., Wang, L. J., Mao, Y. B., Cai, W. J., Xue, H. W., and Chen, X. Y. (2005). Control of root cap formation by MicroRNA-targeted auxin response factors in Arabidopsis. *Plant Cell* 17, 2204–2216. doi:10.1105/tpc.105.033076.

Wang, K. L. C., Yoshida, H., Lurin, C., and Ecker, J. R. (2004). Regulation of ethylene gas biosynthesis by the Arabidopsis ETO1 protein. *Nature* 428, 945–950. doi:10.1038/nature02516.

Wang, L., Hua, D., He, J., Duan, Y., Chen, Z., Hong, X., et al. (2011). Auxin Response Factor2 (ARF2) and Its Regulated Homeodomain Gene HB33 Mediate Abscisic Acid Response in Arabidopsis. *PLoS Genet.* 7, e1002172. doi:10.1371/journal.pgen.1002172.

Wang, Y., Shen, W., Chan, Z., and Wu, Y. (2015). Endogenous cytokinin overproduction modulates ROS homeostasis and decreases salt stress resistance in Arabidopsis thaliana. *Front. Plant Sci.* 6. doi:10.3389/fpls.2015.01004.

Wang, Z. Y., Nakano, T., Gendron, J., He, J., Chen, M., Vafeados, D., et al. (2002). Nuclear-localized BZR1 mediates brassinosteroid-induced growth and feedback suppression of brassinosteroid biosynthesis. *Dev. Cell* 2, 505–513. doi:10.1016/S1534-5807(02)00153-3.

Wasternack, C., and Hause, B. (2013). *Jasmonates: Biosynthesis, perception, signal transduction and action in plant stress response, growth and development. An update to the 2007 review in Annals of Botany*. Oxford Academic doi:10.1093/aob/mct067.

Wasternack, C., and Song, S. (2017). Jasmonates: Biosynthesis, metabolism, and signaling by proteins activating and repressing transcription. *J. Exp. Bot.* 68, 1303–1321. doi:10.1093/jxb/erw443.

Watanabe, S., Sato, M., Sawada, Y., Tanaka, M., Matsui, A., Kanno, Y., et al. (2018). Arabidopsis molybdenum cofactor sulfurase ABA3 contributes to anthocyanin accumulation and oxidative stress tolerance in ABA-dependent and independent ways. *Sci. Rep.* 8, 16592. doi:10.1038/s41598-018-34862-1.

Waters, M. T., Gutjahr, C., Bennett, T., and Nelson, D. C. (2017). Strigolactone Signaling and Evolution. *Annu. Rev. Plant Biol.* 68, 291–322. doi:10.1146/annurev-arplant-042916-040925.

Werner, T., Motyka, V., Laucou, V., Smets, R., Van Onckelen, H., and Schmülling, T. (2003). The Plant Cell Cytokinin-Deficient Transgenic Arabidopsis Plants Show Multiple Developmental Alterations Indicating Opposite Functions of Cytokinins in the Regulation of Shoot and Root Meristem Activity. *Plant Cell* 15, 2532–2550. doi:10.1105/tpc.014928.

Werner, T., Motyka, V., Strnad, M., and Schmülling, T. (2001). Regulation of plant growth by cytokinin. *Proc. Natl. Acad. Sci. U. S. A.* 98, 10487–10492. doi:10.1073/pnas.171304098.

Werner, T., and Schmülling, T. (2009). Cytokinin action in plant development. *Curr. Opin. Plant Biol.* 12, 527–538. doi:10.1016/j.pbi.2009.07.002.

Xie, Q., Essemine, J., Pang, X., Chen, H., and Cai, W. (2020). Exogenous application of abscisic acid to shoots promotes primary root cell division and elongation. *Plant Sci.* 292, 110385. doi:10.1016/j.plantsci.2019.110385.

Xing, L., Zhao, Y., Gao, J., Xiang, C., and Zhu, J. K. (2016). The ABA receptor PYL9 together with PYL8 plays an important role in regulating lateral root growth. *Sci. Rep.* 6, 1–13. doi:10.1038/srep27177.

Yamada, M., Greenham, K., Prigge, M. J., Jensen, P. J., and Estelle, M. (2009). The *TRANSPORT INHIBITOR RESPONSE2* Gene Is Required for Auxin Synthesis and Diverse Aspects of Plant Development. *Plant Physiol.* 151, 168–179. doi:10.1104/pp.109.138859.

Yan, J., Zhang, C., Gu, M., Bai, Z., Zhang, W., Qi, T., et al. (2009a). The arabidopsis CORONATINE INSENSITIVE1 protein is a jasmonate receptor. *Plant Cell* 21, 2220–2236. doi:10.1105/tpc.109.065730.

Yan, Z., Zhao, J., Peng, P., Chihara, R. K., and Li, J. (2009b). BIN2 functions redundantly with other arabidopsis GSK3-like kinases to regulate brassinosteroid signaling. *Plant Physiol.* 150, 710–721. doi:10.1104/pp.109.138099.

Yang, W., Zhang, W., and Wang, X. (2017). *Post-translational control of ABA signalling: the roles of protein phosphorylation and ubiquitination*. Blackwell Publishing Ltd doi:10.1111/pbi.12652.

Zhai, Q., and Li, C. (2019). *The plant Mediator complex and its role in jasmonate signaling*. Oxford University Press doi:10.1093/jxb/erz233.

Zhao, Y. (2012). Auxin biosynthesis: A simple two-step pathway converts tryptophan to indole-3-Acetic acid in plants. in *Molecular Plant* (Oxford University Press), 334–338. doi:10.1093/mp/ssr104.

Zhao, Y., Hull, A. K., Gupta, N. R., Goss, K. A., Alonso, J., Ecker, J. R., et al. (2002). Trp-dependent auxin biosynthesis in Arabidopsis: Involvement of cytochrome P450s CYP79B2 and CYP79B3. *Genes Dev.* 16, 3100–3112. doi:10.1101/gad.1035402.

Zürcher, E., and Müller, B. (2016). “Cytokinin Synthesis, Signaling, and Function-Advances and New Insights,” in *International Review of Cell and Molecular Biology* (Elsevier Inc.), 1–38. doi:10.1016/bs.ircmb.2016.01.001.
